# HMGB1 mediates invasion and PD-L1 expression through RAGE-PI3K/AKT signaling pathway in MDA-MB-231 breast cancer cells

**DOI:** 10.1186/s12885-022-09675-1

**Published:** 2022-05-24

**Authors:** Kamolporn Amornsupak, Suyanee Thongchot, Chanida Thinyakul, Carol Box, Somaieh Hedayat, Peti Thuwajit, Suzanne A. Eccles, Chanitra Thuwajit

**Affiliations:** 1 Department of Transfusion Medicine and Clinical Microbiology, Faculty of Allied Health Sciences, Chulalongkorn University, Bangkok, 10330 Thailand; 2 Immunomodulation of Natural Products Research Group, Faculty of Allied Health Sciences, Chulalongkorn University, Bangkok, 10330 Thailand; 3 Department of Immunology, Faculty of Medicine Siriraj Hospital, Mahidol University, Bangkok, 10700 Thailand; 4 Siriraj Center of Research Excellence for Cancer Immunotherapy, Research Department, Faculty of Medicine Siriraj Hospital, Mahidol University, Bangkok, 10700 Thailand; 5 Centre For Cancer Imaging, Division of Radiotherapy and Imaging, The Institute of Cancer Research, London, SW7 3RP UK; 6 Present Address: Cancer Research UK, Cancer Therapeutics Unit, The Institute of Cancer Research, London, SW7 3RP UK

**Keywords:** Breast cancer, HMGB1, RAGE, PI3K-AKT, Migration, Invasion, PD-L1

## Abstract

**Background:**

High-mobility group box 1 (HMGB1) is increased in breast cancer cells as the result of exposure to the secreted substances from cancer-associated fibroblasts and plays a crucial role in cancer progression and drug resistance. Its effect, however, on the expression of programmed death ligand 1 (PD-L1) in breast cancer cells has not been investigated. This study aimed to investigate the mechanism of HMGB1 through receptors for advanced glycation end products (RAGE) on cell migration/invasion and PD-L1 expression in breast cancer cells.

**Methods:**

A 3-dimensional (3-D) migration and invasion assay and Western blotting analysis to evaluate the function and the mechanism under recombinant HMGB1 (rHMGB1) treatment with knockdown of RAGE using sh*RAGE* and PI3K/AKT inhibitors was performed.

**Results:**

The results revealed that rHMGB1 induced MDA-MB-231 cell migration and invasion. The knockdown of RAGE using sh*RAGE* and PI3K/AKT inhibitors attenuated 3-D migration and invasion in response to rHMGB1 compared to mock cells. PD-L1 up-regulation was observed in both parental MDA-MB-231 (P) and MDA-MB-231 metastasis to bone marrow (BM) cells treated with rHMGB1, and these effects were alleviated in RAGE-knock down (KD) breast cancer cells as well as in PI3K/AKT inhibitor-treated cells.

**Conclusions:**

Collectively, these findings indicate that HMGB1-RAGE through PI3K/AKT signaling promotes not only breast cancer cell invasion but also PD-L1 expression which leads to the destruction of the effector T cells. The attenuating HMGB1-RAGE-PI3K/AKT pathway may help to attenuate breast cancer cell aggressive phenotypes.

**Supplementary Information:**

The online version contains supplementary material available at 10.1186/s12885-022-09675-1.

## Background

Breast cancer is a major health problem with a high incidence of new cases [1]. High mobility group box 1 (HMGB1) is a nuclear protein acting as a gene expression regulator intracellularly, but if it is released outside, it can bind to the receptor for advanced glycation end products (RAGE) and induce intracellular signaling pathways [2]. This study has shown that cancer-associated fibroblast substances can activate HMGB1 expression and secretion by cancer cells, which can then be released into the tumor microenvironment and the HMGB1-RAGE interaction promotes breast cancer progression and drug resistance [3]. It remains to be determined whether such a mechanism is indeed happening in breast cancer cells by the action of the HMGB1-RAGE interaction or if this could be a target for therapeutic intervention.

HMGB1-RAGE ligation has been found to activate several signaling pathways in different cancer types [4]. The PI3K/AKT pathway has been reported to be involved in HMGB1 activation in lung cancer, breast cancer, and cutaneous squamous cell carcinoma [5]. The PI3K/AKT dependent signaling pathway was suppressed by HMGB1, silenced in MCF-7 breast cancer cells and caused cancer cells to have less aggressive phenotypic characteristics including migration, invasion, and angiogenesis [6]. Attenuation of RAGE levels is associated with the decrease of NF-κB and MMP-9 activities [7]. The suppression of HMGB1 and RAGE expressions in breast cancer revealed the impaired invasion capability without affecting cell proliferation, however, this unclear mechanism controlling this effect needs to be investigated [8].

Programmed death ligand 1 (PD-L1) is an immune checkpoint molecule which when binding with the PD-1 receptor can inhibit the effector functions of T lymphocytes. PD-L1 is commonly found to be aberrantly overexpressed in several cancers including breast cancer [9]. Immune checkpoint inhibitors and in particular PD-L1 inhibitors, have been approved for treatment in patients suffering from the advanced stage of triple negative breast cancer [10]. Recently, HMGB1 that is secreted by melanocytes and keratinocytes upon ultraviolet radiation, activated NF-κB- and IRF3-dependent transcription of PD-L1 in melanocytes through RAGE [11]. HMGB1-mediated PD-L1 expression in breast cancer through the PI3K/AKT signaling pathway, however, has not been investigated. The cancer cells having high PD-L1 are resistant to anti-tumor T cell responses, hence the high expression of PD-L1 in cancer cells induced by cancer-associated fibroblast substances may lead to the progression of cancer.

In this present study, recombinant HMGB1-activated MDA-MB-231 breast cancer cell migration and invasion were investigated. The RAGE-knocked down (KD) breast cancer and inhibitors against PI3K and AKT were applied to investigate that RAGE-PI3K/AKT signaling pathway activated by HMGB1 could regulate aggressive breast cancer cell properties and PD-L1 expression leading to acquired resistance to breast cancer treatment.

## Methods

### Human breast cancer cell lines

MDA-MB-231 parental cells (MDA-MB-231 P) were originally obtained from ATCC-LGC (Middlesex, UK) while MDA-MB-231 that was metastasized to bone marrow (MDA-MB-231 BM) was obtained from the 2^nd^ generation of MDA-MB-231 cells which had metastasized to bone following intracardiac injection of MDA-MB-231 lung metastasis cells as previously reported [12].

### Two-dimensional (2-D) and three-dimensional (3-D) monolayer tumor cell cultures

MDA-MB-231 P and MDA-MB-231 BM cells were routinely cultured in DMEM (Gibco BRL, Grand Island, NY), supplemented with 10% heat-inactivated fetal bovine serum (FBS) (Thermo Fisher Scientific Inc., Carlsbad, CA, USA) and designated as a complete medium. Cells were grown as a monolayer in a sterile culture flask at 37 °C in a humidified incubator with 5% CO_2_.

Tumor spheroids were initiated by seeding 1 × 10^3^ breast cancer cells supplemented with 2.5% cold Matrigel™ (354234, BD Biosciences San Jose, CA, USA) in complete media into pre-cooled 96-well ultra-low attachment (ULA) plates (7007, Costar/Corning Amsterdam, Netherlands). Plates were kept on ice during cell seeding before centrifugation at 4 °C at 200 x *g* for 3-5 min. Spheroids were established for 96 h at 37 °C in a 5% CO_2_ incubator before use for further studies. Treatment or 50% media replenishment and imaging for determining tumor spheroid growth kinetics were performed on days 4, 7, and 10 after tumor spheroid initiation (day 0). The mean radius was used to calculate the volume with the formula 4/3πr^3^. The percentage increase in volume was expressed relative to day 4 (t0).

### Western blot analysis

The 2 × 10^5^ breast cancer cells were seeded in 6-well plates overnight and starved in 0.1% bovine serum albumin (BSA) (Capricorn Scientific GmgH, Ebsdorfergrund, Germany) containing DMEM for 48 h. Then, 25 or 100 ng/ml of rHMGB1 (1690-HMB, R&D Systems, Minneapolis, MN, USA) in 0.1% BSA DMEM or 10% FBS DMEM for the indicted time. For small molecule inhibitor treatment, cells were treated with 100 ng/ml rHMGB1 or in combination with either 150 nM GDC-0941 (Pictilisib; RG-7321, Genentech Inc., South San Francisco, CA, USA) or 2 μM AT13148 (21597, Cayman Chemical, MI, USA) in 0.1% BSA DMEM for 30 min. Cells were lysed in RIPA buffer (sc-24948A, Santa Cruz Biotechnology) containing lysis buffer, PMSF, sodium orthovanadate, and protease inhibitor cocktail. Cell extracts were then separated by 10% SDS-PAGE and transferred onto PVDF membranes (GE Healthcare, Buckinghamshire, UK) and immunoblot was performed as described previously [13]. Membranes were blocked in 5% skim milk containing TBST (TBS containing 0.1% Tween 20) for 1 h at room temperature. The membranes were incubated with the appropriate primary antibody (Table 1) for the indicated time in blocking buffer at 4 °C overnight. Membranes were washed three times with TBST for 5 min each then incubated with HRP-conjugated goat anti-rabbit IgG H&L (ab6721, Abcam, Cambridge, MA, USA) and HRP-conjugated goat anti-mouse IgG, (7076, Cell Signaling Technology, Danvers, MA, USA). The blots were visualized by enhanced chemiluminescence (Thermo Scientific, Rockford, IL, USA) under Gel Document (Syngene, Cambridge, UK). The bands were quantified by ImageJ version 1.48v (NIH, Bethesda, MD, USA).

**Table 1:** Antibodies and optimal staining conditions for Western blot analysis

First antibody	Clone no. / Company	Dilution	Secondary antibody
Anti-p-AKT Ab	9271/ Cell Signaling	1:500	4 °C, overnight
Anti-AKT Ab	9272/ Cell Signaling	1:1000	4 °C, overnight
Anti-p-ERK Ab	9101/ Cell Signaling	1:1000	4 °C, overnight
Anti-ERK Ab	9102/ Cell Signaling	1:1000	4 °C, overnight
Anti-p-mTOR Ab	2971/ Cell Signaling	1:1000	4 °C, overnight
Anti-mTOR Ab	9272/ Cell Signaling	1:1000	4 °C, overnight
Anti-p-S6 Ab	2215/ Cell Signaling	1:1000	4 °C, overnight
Anti-S6 Ab	2217/ Cell Signaling	1:1000	4 °C, overnight
Anti-p-STAT3 Ab	9131/ Cell Signaling	1: 1000	4 °C, overnight
Anti-STAT3 Ab	9139/ Cell Signaling	1:1000	4 °C, overnight
Anti-p-GSK-3β Ab	9336/ Cell Signaling	1:1000	4 °C, overnight
Anti-GSK-3β Ab	9315/ Cell Signaling	1:1000	4 °C, overnight
Anti-p-JNK Ab	9251/ Cell Signaling	1:1000	4 °C, overnight
Anti-JNK Ab	9252/ Cell Signaling	1:1000	4 °C, overnight
Anti-p-p38 Ab	9211/ Cell Signaling	1:1000	4 °C, overnight
Anti-p38 Ab	9212/ Cell Signaling	1:1000	4 °C, overnight
Anti-PD-L1 Ab	Ab205921/ Abcam	1:500	4 °C, overnight
Anti-β-actin Ab	Sc-47778/ Santa Cruz	1:10,000	4 °C, overnight

### Transduction of RAGE knocked-down breast cancer cells by shRAGE

The breast cancer cells were seeded at 5 × 10^5^ per well into 6-well plates and allowed to reach 80% confluence overnight. Then 100 μM genistein in 10% FBS DMEM was added for at least 4 h [14, 15]. Viral supernatant produced by transfection HEK293T cells with MISSION® sh*RAGE* lentiviral plasmid DNA (Sigma-Aldrich Corporation, St. Louis, MO, USA) or MISSION® TRC2 pLKO.5-puro Empty Vector Control plasmid DNA (Sigma-Aldrich) was used for establishing RAGE-knocked down (RAGE-KD) cells or mock control cells. Viral supernatant was then added with 8 μg/ml polybrene. Cells were centrifuged for 30 min at 800 x *g* and incubated at 37 °C overnight. Fresh DMEM containing 10% FBS was replenished after the spent media was changed to remove the virus. Transduced cells were then passaged after 48 h transduction and cultured in 10% FBS DMEM containing 1 μg/ml puromycin (A1113803, Invitrogen Corporation, Carlsbad, CA, USA) for selection.

### Two-dimensional (2-D) migration assay

The breast cancer cells at 80-90% confluence were starved in serum-free DMEM for 24 h. Cells were fluorescently labeled by incubation with 5 μmol/L Green 5 chloromethyl fluoresceindiacetate CellTracker (Green CMFDA Dye, C2925, Invitrogen, Paisley, Waltham, MA, USA) for 2 h before trypsinization. Cells were counted and 5 × 10^4^ cells/well in 1% BSA DMEM were added into the upper wells of 8 μm-pore Fluoroblok™ membrane inserts in 24-well companion plates (BD Biosciences, Franklin Lakes, NJ, USA). The lower chamber was filled with 800 μl of medium containing 5% FBS as a positive control, 100 ng/ml rHMGB1 in 1% BSA DMEM or 1% BSA DMEM as a negative control. The assay plates were incubated in a humidified atmosphere at 37 °C in a 5% CO_2_ incubator. Imaging was performed at 24 h intervals for up to 48 h. Cells that successfully migrated to the lower surface of the filter were visualized using an inverted fluorescence microscope (Olympus LX70, Olympus, Middlesex, UK). Five different fields of microscopic detection were scored for each well and the cells were counted using Image Pro-Plus 6.3 software (Media Cybernetics, Silver Spring, MD, USA).

### Two-dimensional (2-D) invasion assay

The breast cancer cells were fluorescently labeled by incubation with 5 μmol/L Green CMFDA Dye (Invitrogen) for 2 h. Fluoroblok™ membrane inserts (8 μM pore size, BD Biosciences) were coated with 100 μl of 10% Matrigel™ (BD Biosciences) in cold serum-free DMEM. Companion plates with coated inserts were incubated at 37 °C for 2 h. Then, the remaining liquid (coating buffer) from the permeable support membranes was carefully removed without disturbing the layer of Matrigel™ (BD Biosciences) on the membranes. After 2 h, cells were counted and 5 × 10^4^/well in 1% BSA DMEM without or with 100 ng/ml rHMGB1 (R&D Systems) or in a combination with either 150 nM GDC-0941 or 2 μM AT13148 was added into the upper wells of coated 8-μm pore Fluoroblok™ membrane inserts in 24-well companion plates (BD Biosciences). The lower chamber was filled with 800 μl medium containing 5% FBS DMEM. The assay plates were incubated in a humidified atmosphere at 37 °C in a 5% CO_2_ incubator. Imaging was performed as described in the chemotaxis assay.

### Tumor spheroid-based migration assay

Spheroids were created by 1 × 10^3^ breast cancer cells supplemented with cold Matrigel™ (BD Biosciences) in 200 μl of complete DMEM medium and seeded into individual wells of flat, clear-bottomed, black-walled polystyrene 96-well plates (655090, Greiner bio one, Merck KGaA, Darmstadt, Germany) that were pre-coated with 100 μl/well of 0.1% (v/v) gelatin type B (G1393, Sigma-Aldrich) in sterile water for at least 1 h at 37 °C. Excess gelatin was removed and plates were washed with serum-free DMEM. Then, 200 μl/well of human rHMGB1 (R&D Systems) at various concentrations in 0.1% BSA DMEM were added. DMEM with 0.1% BSA and DMEM with 2% FBS were used as negative and positive controls. Four-day-old spheroids were then carefully transferred from the 96-well ULA plates, used for spheroid initiation, into the center of each gelatin-coated well of the prepared migration plates. Spheroids were allowed to adhere to the gelatin for 1 h at 37 °C prior to imaging at intervals. The data were normalized to the area of each spheroid recorded at t_0_.

### Tumor spheroid-based invasion assays

A total of 100 μl medium was carefully removed from wells containing 4-day old spheroids and the plates were then placed on ice for 10 min to cool. Matrigel (with or without human rHMGB1 or 10% FBS) was diluted 1:2 with ice-cold 0.1% BSA DMEM and 100 μl was gently added to each well. Plates were centrifuged at 300 x *g* for 3 min at 4 °C and then incubated at 37 °C for 2 h. The 100 μl of 0.1% BSA DMEM (with or without human rHMGB1 or 10% FBS) was added on top. Images of cells invading spheroids from t_0_ and at intervals up to 96 h as well as data analysis of the extent of invasion were produced. The area of each spheroid was analyzed and expressed as a percentage of the spheroid area on day 4 (t_0_) with the formula: % cell invasion = [invaded area at t_x_/spheroid area at t_0_] × 100).

### Statistical analysis

The values were expressed as mean ± standard deviation (SD). Statistical significance was determined by Student’s *t*-test and one-way ANOVA followed by Tukey’s post-hoc test. Statistical analyses were performed using SPSS 20.0 statistics software (SPSS, IBM, Armonk, NY, USA). A *P*-value of less than 0.05 was defined as statistically significant.

## Results

### HMGB1-activated signaling pathway in breast cancer cell lines

The Western blot results showed that all breast cancer cell lines had similar basal levels of RAGE (Supplementary Fig. 1). MDA-MB-231 BM had higher p-AKT than the parental subline. In addition, p-ERK expression was high in all MDA-MB-231 sublines (Supplementary Fig. 1). The rHMGB1 induced p-AKT in a time-dependent manner (Supplementary Fig. 2) with a maximal level after 30 min post-rHMGB1 treatment. Moreover, p-S6 and p-ERK were detected at 20 min and 30 min after rHMGB1 treatment. No increased levels of p-JNK, p-GSK-3β, p-STAT3, and p-p38, however, were observed in MDA-MB-231 P cells compared to normal controls without rHMGB1 treatment (Supplementary Fig. 2). The densitometry results revealed the increase of only p-AKT at the suitable time and dose of rHMGB1, hence, it was selected to be investigated in the study.

At 30 min post-rHMGB1 treatment, the results showed that the level of p-AKT increased in a dose-dependent manner in MDA-MB-231 P and 100 ng/ml was the optimal concentration for p-AKT activation (Supplementary Fig. 2). Moreover, increased levels of p-ERK1/2 and p-mTOR were detected in MDA-MB-231. p-JNK, p-GSK-3β, p-STAT3, and p-p38, however, showed no increase compared to the control condition (Supplementary Fig. 2). No changes of either p-AKT or p-ERK1/2 were detected in rHMGB1-treated MDA-MB-231 BM cells (Supplementary Fig. 3).

### Effect of rHMGB1 on tumor cell proliferation, migration, and invasion

**Fig. 1:**
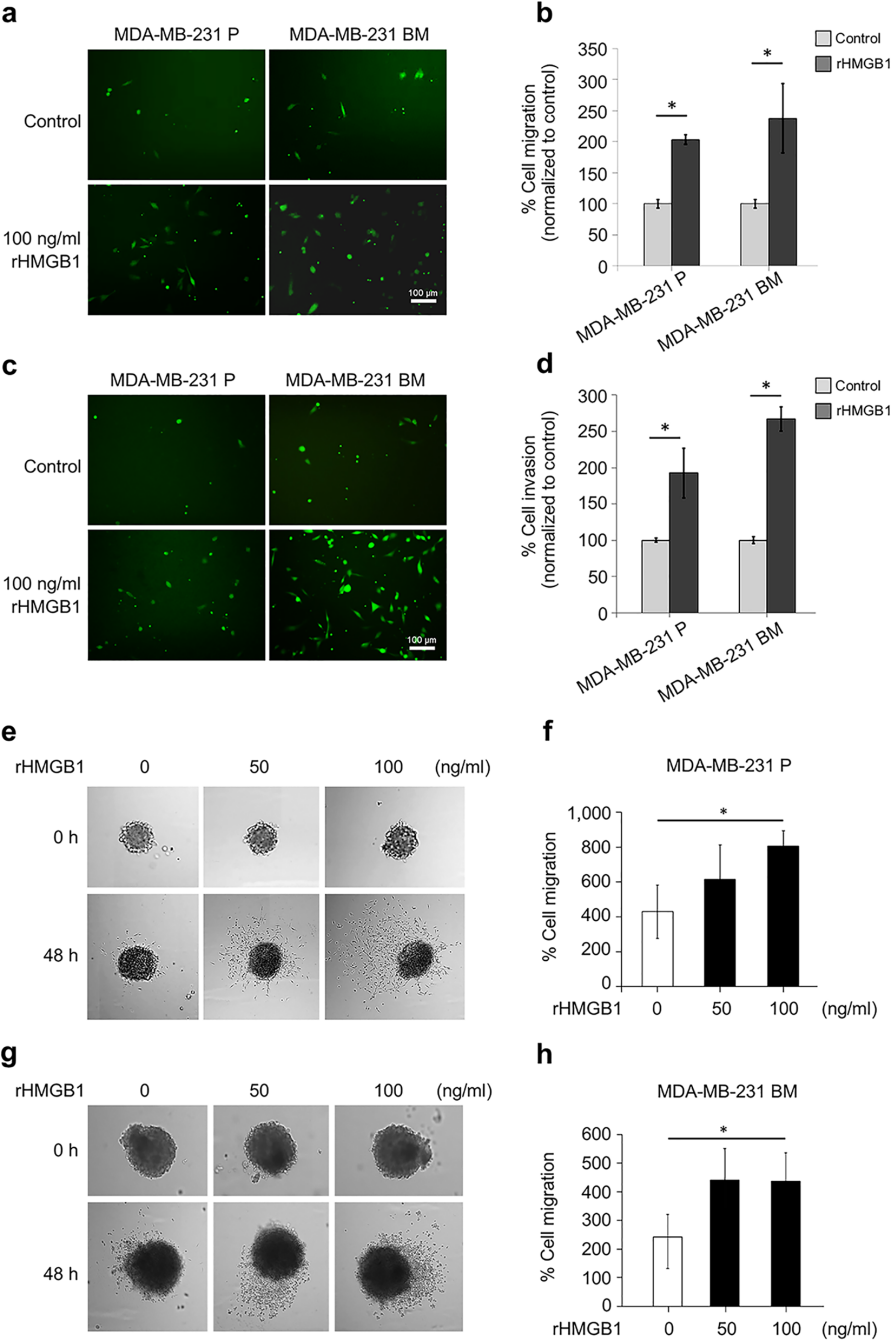
The effects of rHMGB1 on breast cancer migration and invasion. Chemo-migration was tested in a Fluoroblok™ Transwell migration assays for MDA-MB-231 P and MDA-MB-231 BM with 100 ng/ml rHMGB1 in 1% BSA DMEM as the chemoattractant. Representative images of cells which successfully migrated to the lower surface of the membrane were visualized using an inverted fluorescence microscope are shown (**a**). Bar graphs that represent migration of control (1% BSA DMEM) calculated from the number of migrated cells of 10 images taken from 2 wells per conditions (**b**). Cell invasion was tested in a Matrigel-coated Transwell invasion assay for MDA-MB-231 P and MDA-MB-231 BM with 100 ng/ml rHMGB1 in 1% BSA DMEM for 48 h. Representative images of cells that successfully invaded the lower surface of the insert visualized using an inverted fluorescence microscope are shown (**c**). Bar graphs represent percent invasion of controls unstimulated (1% BSA DMEM) calculated from the number of invaded cells of 10 images taken from 2 wells as per conditions (**d**). Results are presented as mean ± SD of duplicate independent experiments. Scale bar = 100 μm and original magnification 100X. For 3-D tumor spheroid-based migration assay. Tumor spheroids of MDA-MB-231 P and MDA-MB-231 BM were transferred to each well of a 96-well flat-bottomed plate coated with 0.1% gelatin which contained rHMGB1 at various concentrations in 0.1% BSA DMEM. Representative bright-field images of cell migration of MDA-MB-231 P at 48 h (**e** and **f**) and MDA-MB-231 BM at 96 h (**g** and **h**) are shown. Bar graphs represent percent cell migration normalized against the control untreated cells. Results are presented as mean ± SD of duplicate independent experiments. *P < 0.05

**Fig. 2:**
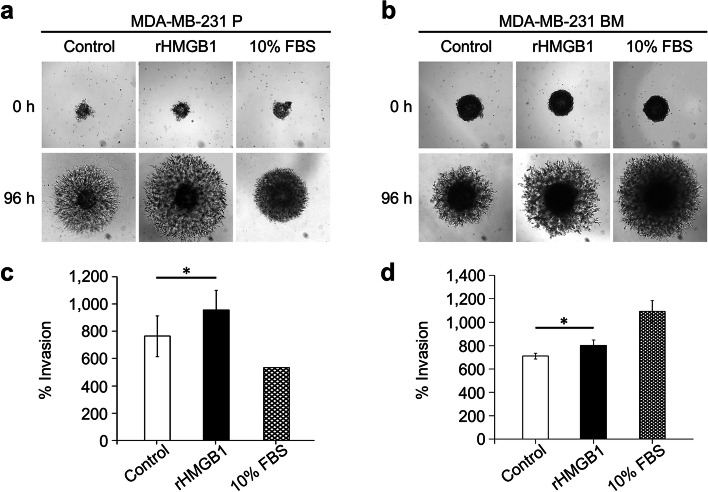
Effect of rHMGB1 on breast cancer cell invasion in a 3-D tumor spheroid-based invasion assay. MDA-MB-231 P cell invasions at 96 h from the tumor spheroid after 100 ng/ml rHMGB1 treatment (**a** and **c**) and MDA-MB-231 BM (**b** and **d**). Representative bright-field images of cell migration at 0 h and 96 h are shown. Graphs represent percent cell invasion of control unstimulated cells (0.1% BSA DMEM). Bars represent mean ± SD of duplicate independent experiments. *P < 0.05

The role of HMGB1 on cell proliferation in breast cancer cells was investigated by 3-D tumor spheroid-based assays. The results showed that no effect of rHMGB1 on cell proliferation was observed in MDA-MB-231 P tumor spheroids compared to the control rHMGB-untreated cells at all doses (Supplementary Fig. 4). The rHMGB1 significantly induced cell migration in MDA-MB-231 P and MDA-MB-231 BM cells more than the untreated control cells (Fig. 1a and b). The invasion assay results revealed that rHMGB1 significantly induced cell invasion in MDA-MB-231 P and MDA-MB-231 BM more than 1% BSA DMEM control (Fig. 1c and d). Furthermore, cell migration from tumor spheroids was significantly increased in MDA-MB-231 P tumor spheroids treated with 100 ng/ml rHMGB1 compared to the control conditions after 48 h (Fig. 1e and f). This effect was also observed in MDA-MB-231 BM cells after 100 ng/ml rHMGB1 treatment for 96 h (Fig. 1g and h). In 3-D spheroid-based-invasion assay, the results revealed the increase in cancer cell invasion from the tumor spheroids was significantly detected in MDA-MB-231 P and MDA-MB-231 BM (Fig. 2).

### Effect of RAGE silencing and AKT inhibitors on HMGB1-mediated breast cancer cell invasion

**Fig. 3:**
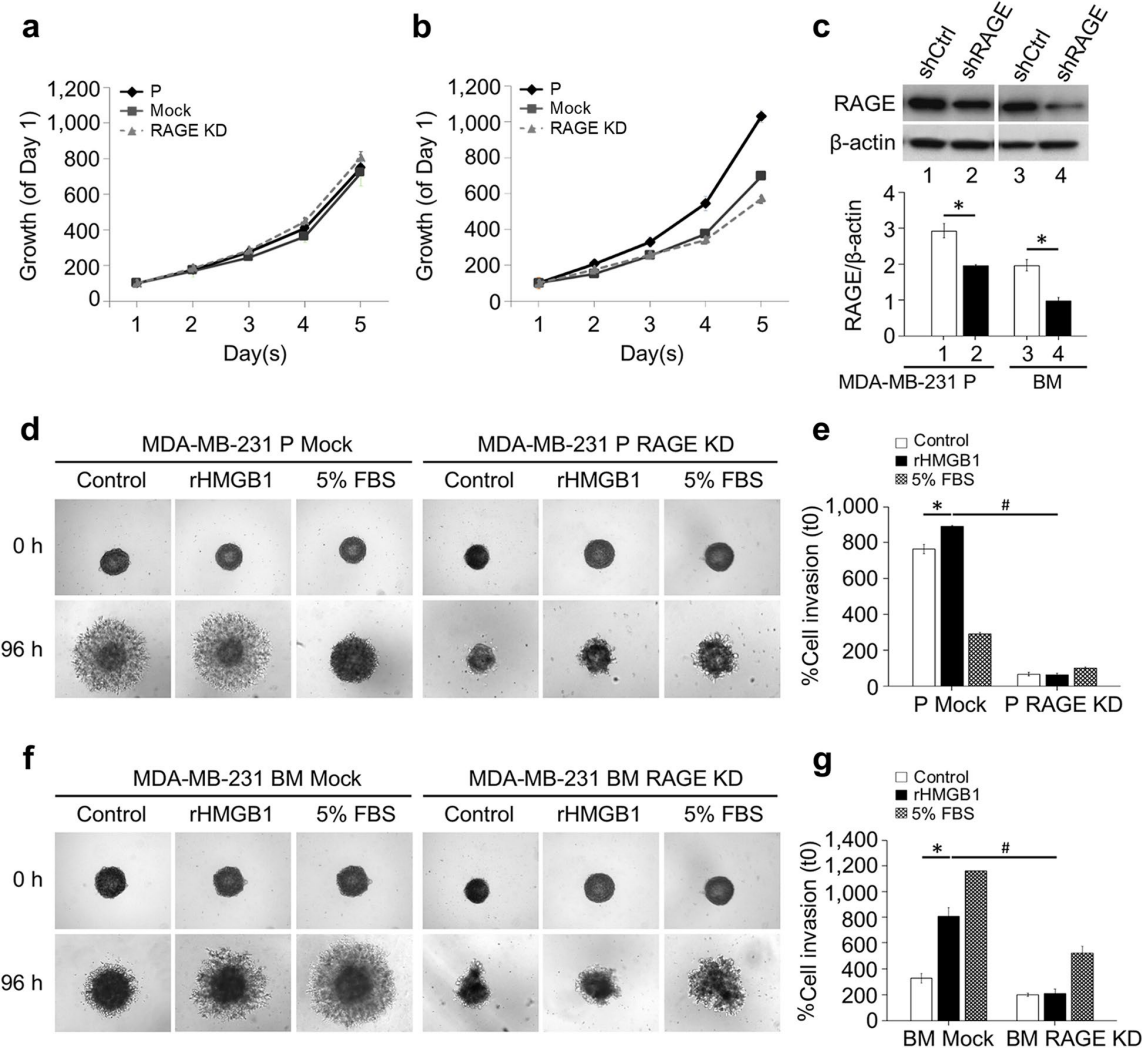
Effect of RAGE silencing on rHMGB1-mediated tumor cell invasion. 3-D growth of parental, mock, RAGE-KD cells of MDA-MB-231 P and MDA-MB-231 BM (**a** and **b**). Graphs represent the average of six data sets for each condition. The expression levels of RAGE protein in mock and RAGE-KD of MDA-MB-231 P and MDA-MB-231 BM cells was detected by Western blot analysis (**c**). Representative images of tumor spheroid invasion after 100 ng/ml rHMGB1 treatment of MDA-MB-231 P (**d** and **e**) and MDA-MB-231 BM (**f** and **g**) are shown. Bars represent mean ± SD of duplicate independent experiments. *P < 0.05; comparing % cell invasion of mock-treated cells with or without 100 ng/ml rHMGB1; ^#^*P* < 0.05; comparing % cell invasion of rHMGB1 treated cells in mock-treated and RAGE-KD cells

The results exhibited that RAGE*-*KD reduced expression of the receptor to around 50% of the basal levels with no effect on cell growth (Fig. 3a and b). Western blot results revealed that the RAGE level was decreased under sh*RAGE* treatment in both MDA-MB-231 P and MDA-MB-231 BM cells leading to 30 and 50% efficiency knock-down of RAGE (Fig. 3c). The results showed that rHMGB1 induced cell invasion was significantly attenuated in RAGE-KD MDA-MB-231 cells in the 3-D-spheroid invasion assay. The decrease of RAGE reduced cell invasion in HMGB1-treated MDA-MB-231 P cells (Fig. 3d and e). Similar results were observed in RAGE-KD MDA-MB-231 BM cells (Fig. 3f and g).

**Fig. 4:**
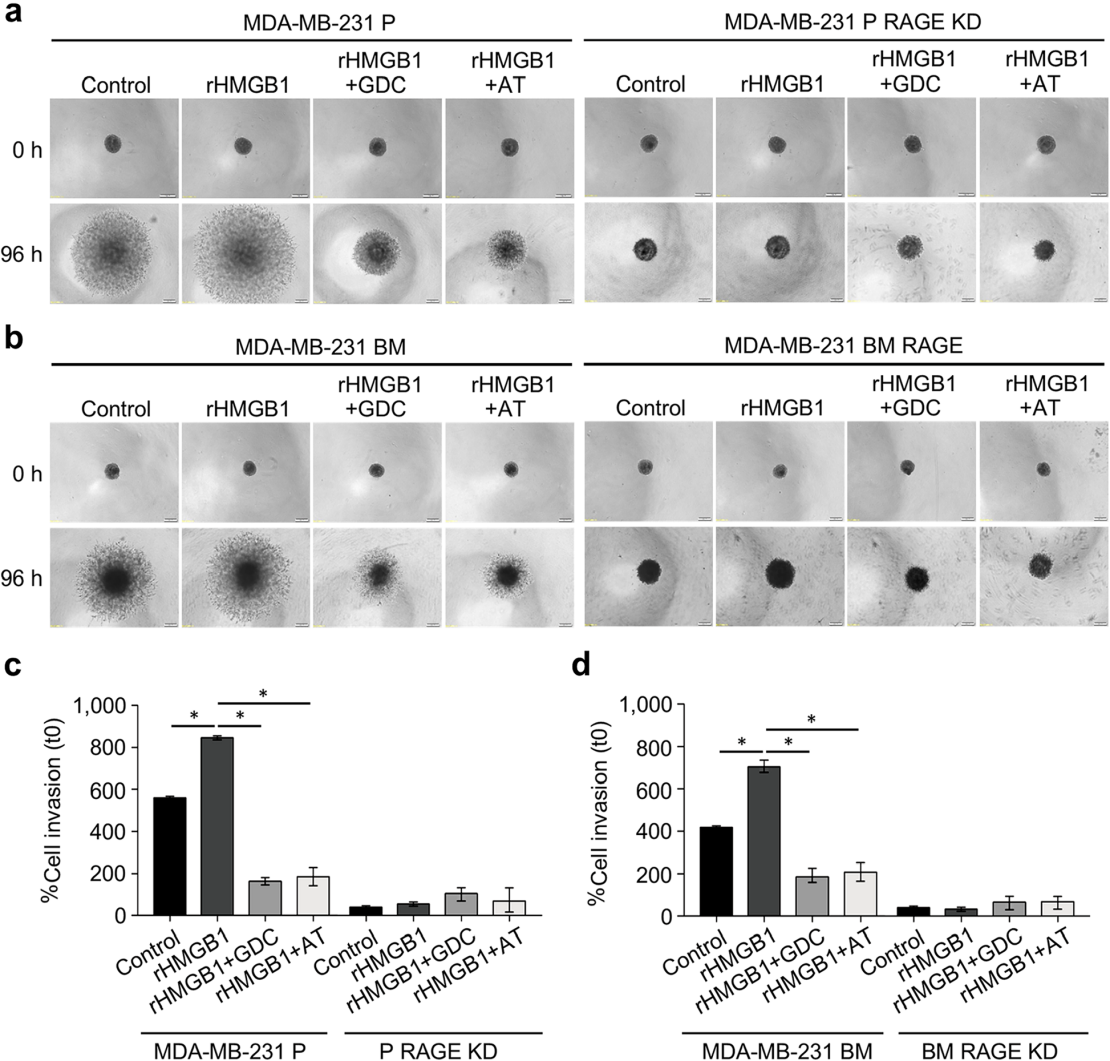
Effects of PI3K inhibitor and AKT inhibitor on cell invasion in RAGE KD breast cancer cell lines. Cell invasion was determined with 100 ng/ml rHMGB1 or in a combination with either 150 nM GDC-0941 or 2 μM AT13148 in 1% BSA DMEM for 48 h. Representative images of invaded cells were visualized using an inverted fluorescence microscope, scale bar = 200 μm, and original magnification 40X (**a** and **b**). Bar graphs represent percent cell invasion of unstimulated controls (1% BSA DMEM) calculated from the number of invaded cells of 10 images taken from 2 wells per conditions (**c** and **d**). *P < 0.05

To confirm, PI3K/AKT as the dependent pathway activated by HMGB1, specific inhibitors, GDC-0941 and AT13148, were used. The SRB assay revealed the IC_50_ values of GDC-0941 and AT13148 were 150 nM and 2 μM for both MDA-MB-231 P and MDA-MB-231 BM cells (Supplementary Fig. 5). No morphological changes were observed when cells were exposed to the inhibitors. As expected, the invasive capability of both MDA-MB-231 P and MDA-MB-231 BM cells was inhibited upon GDC-0941 and AT13148 treatment, whereas these effects were not observed in RAGE deficient cells (Fig. 4a-d).

### Effect of RAGE-KD and PI3K/AKT inhibitors in HMGB1-mediated PD-L1 expression

**Fig. 5:**
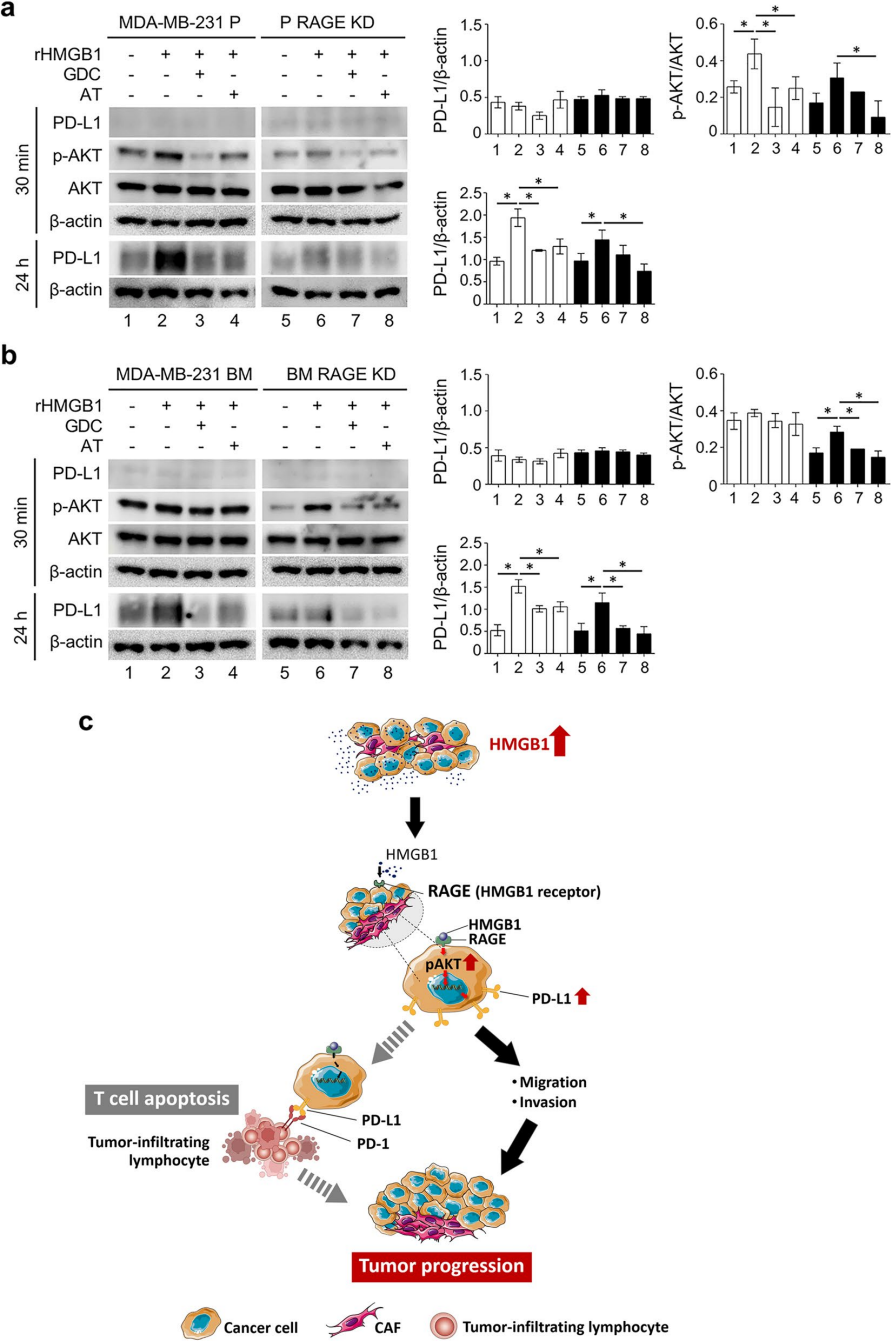
PI3K signaling pathway activation by rHMGB1. MDA-MB-231 P and MDA-MB-231 BM cells were starved in 0.1% BSA DMEM for 48 h, then treated with 100 ng/ml rHMGB1 or in combination with either 150 nM GDC-0941 or 2 μM AT13148 in 0.1% BSA DMEM for 30 min and 24 h (**a** and **b**). Relative protein expression levels are of p-AKT normalized by AKT and PD-L1 normalized by β-actin. Results are presented as mean ± SD of three independent experiments. Data are expressed as mean ± SD of four experiments. *P < 0.05. The schematic diagram illustrates the potential of the RAGE-PI3K/AKT signaling pathway in which HMGB1 regulates breast cancer cell aggressiveness and PD-L1 expression. T cells can use PD-1 to ligate with PD-L1 on cancer cells leading to T cell apoptosis (**c**). The dark lines represent the data in this study

The immunoblot analysis showed the basal levels of p-AKT and AKT were detected at 30 min whereas PD-L1 expression was detected at 24 h (Fig. 5a and b). The transfection with the specific shRNA to *RAGE* resulted in efficient down-regulation of AKT in both breast cancer cell lines. These data suggested PI3K inhibitor GDC-0941 abolished phosphorylation of AKT in all cell lines, especially in MDA-MB-231 P cells and RAGE KD-MDA-MB-231 P cells (Fig. 5a and b). Notably, p-AKT levels of both cells were increased upon AT13148 treatment and was lower than in the rHMGB1 stimulated condition (Fig. 5).

The PD-L1-mediated PI3K/AKT pathway was observed after 100 ng/ml rHMGB1 treatment for 24 h in MDA-MD-231 P, RAGE KD-MDA-MD-231 P, MDA-MB-231 BM, and RAGE KD-MDA-MB-231 BM cells (Fig. 5a and b). Through PI3K and AKT inhibitors under rHMGB1 treatment at 24 h, PD-L1 constituted a functional link between PI3K/AKT and aggressive activity of cancer cells. Two inhibitors (GDC0941 and AT13148) could largely prevent the expression of PD-L1 in all four cell lines. Some leftovers of PD-L1 were present in sh*RAGE*-transfected MDA-MB-231 P and MDA-MB-231 BM cells under GDC-0941 and AT13148 treatment when compared with MDA-MB-231 P and MDA-MB-231 BM (24 h). Taken all together, these data suggest that the HMGB1-RAGE-mediated PI3K/AKT pathway led to mediate PD-L1 expression in breast cancer cells.

## Discussion

HMGB1 has several important roles in inflammation and cancer and appears to play paradoxical roles during the development and therapy of cancer according to subcellular locations, receptors, and expression levels [16]. Increased HMGB1 expressions are associated with each of the hallmarks of cancer including sustained angiogenesis, evading apoptosis, self-sufficiency in growth signals, insensitivity to inhibitors of growth, inflammation, tissue invasion, and metastasis. Although the overexpression of HMGB1 in breast cancer has been increasingly reported, the role of HMGB1 in breast cancer is still controversial due to its conflicting tumor-promoting and tumor-suppressive roles. The macrophage migration inhibitory factor could induce breast cancer cell migration and metastasis via HMGB1/TLR4/NF-κB activation [17]. Moreover, HMGB1 silenced attenuated migration and invasion abilities of the MCF-7 cell line [18]. In the present study, HMGB1 treatment of breast cancer cells with intact or deficient RAGE expression revealed the ligation of HMGB1-RAGE through PI3K/AKT signaling pathways leading to cancer cell migration and invasion. The inhibitors of PI3K and AKT confirm these findings. Additionally, this signaling pathway activates the presence of PD-L1 in breast cancer cells.

The extracellular effect of HMGB1 on cancer cells in 2-D culture of the breast cancer cell model showed enhanced cancer cell migration and invasion. The degree of migration and invasion induced by rHMGB1 were correlated with the aggressiveness of cell lines. No effect of extracellular HMGB1 on cell proliferation, however, was observed in these cell lines. The HMGB1 mediation of the PI3K/AKT pathway in MCF-7 cells [6] was modulated by its intracellular role. The role of extracellular HMGB1 on breast cancer progression via promoting cancer cell migration and invasion herein was supported by the recent reports showing that downregulation of miR-205 contributes to epithelial-mesenchymal transition (EMT) and invasion in triple-negative breast cancer by targeting the HMGB1-RAGE signaling pathway [19].

RAGE was bound by several ligands which activate three main signaling pathways including JAK/STAT, PI3K/AKT, and MAPK/ERK [20]. The HMGB1 and RAGE ligation activates EMT phenomenon and induces tumor growth in breast cancer [19]. Therapeutic strategies to block RAGE may represent great therapeutic potential and therefore it has been under extensive investigation during the last decade. Hence, it is of great interest for several research groups to explain the mechanisms of how HMGB1/RAGE signaling pathway controls these tumorigenic properties.

Adherent cell monolayer culture is an inadequate representation of *in vivo* tumor biology due to the lack of microenvironmental signals originating from cell-cell or cell-substrate interactions. Therefore, 3-D cultures, which better mirrors the in vivo tumor microenvironment in terms of cellular heterogeneity, nutrient and oxygen gradients, cell-cell interactions, and matrix deposition [21], is the method of interest to explore the effect of extracellular HMGB1. Similar results were observed in 3-D spheroid migration and invasion assays which revealed that rHMGB1 promoted cancer cell migration in all three cell lines and induced cancer cell invasion in the MDA-MB-231 P and BM sublines. Of note, no invasion was observed in MCF-7 tumor spheroids even in positive control conditions which could be explained by the weak malignant potential of this cell line (data not shown). Moreover, this could be explained by the evidence showing that 3-D culture decreased invasive potential of MCF-7 cells by increasing E-cadherin, tight-junctions, and desmosomes leading to a more cohesive structure of these spheroids [22]. In addition, invasion from the spheroid observed in MDA-MB-213 P and BM sublines cultured in basal conditions could be explained by the possible presence of the autocrine function of the secreted HMGB1 from cancer cells inside the spheroid where hypoxia and glucose deprivation are presented (data not shown). These findings demonstrated that the extracellular role of HMGB1 on breast cancer progression is probably through promoting tumor cell migration and invasion as well as the acquired doxorubicin resistance via p-ERK mediated autophagy [23].

Blockade of HMGB1-RAGE interaction by soluble or mutated RAGE resulted in suppression of tumor growth and metastasis in glioma. Moreover, transient knockdown of RAGE suppressed the cellular proliferation and invasive activity of hepatocellular carcinoma cells together with the decreased NF-κB expression [7]. Furthermore, the binding of HMGB1 to RAGE, but not TLR4, could induce Beclin1-dependent autophagy and promote pancreatic and colon cell line resistance to chemotherapeutic drugs including oxaliplatin, melphalan, Adriamycin, and paclitaxel through autophagic induction [24]. In the present study, decreased RAGE expression by shRNA silencing at a level that did not affect the growth rate of breast cancer cells, dramatically reduced the invasion of breast cancer cells induced by rHMGB1. This is parallel to the finding reported by Kwak T and colleagues that RAGE-silenced breast cancer cells led to the decreased invasion and soft agar colony formation, without affecting proliferation [8]. It is noteworthy that the decrease in the invasive ability of RAGE-knocked down breast cancer cells was also observed under positive control conditions. This could be explained by the fact that RAGE mediated S100A4-induced cell motility via MAPK/ERK signaling pathway and promoted human colorectal cancer metastasis [25]. Moreover, the binding of RAGE to S100A8/A9 promoted the migration and invasion of human breast cancer cells through actin polymerization and EMT. In addition, AGEs-RAGE interaction increased MDA-MB-231 cell proliferation, migration, and invasion together with an enhancement of MMP-9 activities [26]. Therefore, such responses are, of course, dependent on the particular repertoire of these receptors expressed by the cells, which are factors beyond the scope of this study. Hence, from the findings herein, it can be concluded that the pathways in breast cancer by which extracellular HMGB1 promotes disease aggressiveness are mainly through invasion via RAGE.

HMGB1 has a high affinity for RAGE and RAGE signaling can activate two major pathways which are cellular migration and activation of the Rho GTPases/GTPases Rac and CDC42 and diverse Ras-mitogen activated protein kinases (MAPKs) (ERK1/2, p38, and SAPK/JNK) that finally lead to NF-κB-dependent transcriptional activity [27]. Moreover, HMGB1 promoted hepatocellular carcinoma progression partly by enhancing the ERK1/2 and NF-κB pathways, upregulating MMP-2, and downregulating p21 via an ERK/c-Myc pathway [28]. Although the pathways activated by extracellular HMGB1-RAGE interaction in breast cancer cells have not been identified, this study showed that rHMGB1 induced the phosphorylation of AKT, ERK, mTOR, and S6 in MDA-MB-231 P. Only p-AKT but not p-ERK, however, was activated in MCF-7 after rHMGB1 treatment. No changes of either p-AKT or p-ERK1/2 were observed in MDA-MB-231 BM cells. Similar findings reported that levels of p-AKT and p-ERK were increased in MDA-MB-231 P cells treated with rHMGB1 under hypoxic conditions together with nuclear accumulation of NF-κB [29]. Different patterns of signaling molecules after rHMGB1 activation in three breast cancer cell lines may be explained at least, in part, by different intrinsic levels of these signaling molecules in the basal state. Moreover, the different origins of these cells might provide different crosstalk opportunities leading to up-regulation of different patterns of genes and behavior. Taken together, the findings in breast cancer in this study also added to the evidence that HMGB1-RAGE interactions in cancer progression are mainly transduced through PI3K/AKT, MAPKs, and NF-κB signaling pathways.

To investigate the signaling pathways involved in the invasive capability mediated by HMGB1-RAGE interaction, the PI3K inhibitor GDC-0941 and AKT inhibitor AT13148. AT13148, the compound which has been previously reported as a potent antitumor activity [30], were investigated for their effects on human breast cancer herein. The p-AKT levels in both MDA-MB-231 P and MDA-MB-231 BM cells were increased upon AT13148 treatment consistent with a previous report which showed that this induction of phosphorylation does not activate downstream targets and tumor cell growth [30]. Nevertheless, AT13148 could abolish the rHMGB1-induced invasive capability of MDA-MB-231 P and BM cells. This phenomenon may be a consequence of the effect of ROCK inhibition by AT13148, which was previously reported as the side effect of this compound that does not activate downstream targets and tumor cell growth [30]. Consistent with an earlier study that showed an increase of cell invasion in MCF-7 cells upon ROCK inhibitor treatment while another study demonstrated the opposite effect of ROCK inhibition on MDA-MB-231 cells [31]. Notably, in the current study it was shown that PI3K and AKT inhibitors could inhibit rHMGB1-mediated cell invasion in MDA-MB-231 by the functional assay of cell invasion, confirming HMGB1/PI3K/AKT signaling-mediated cell invasion in MDA-MB-231 P. In MDA-MB-231 BM cells, it was observed that the same effect of PI3K inhibitor as in MDA-MB-231 occurred. Thus, it is likely that HMGB1-RAGE interaction promotes breast cancer cell invasion via PI3K/AKT signaling.

PD-L1 prominently activated the EMT process in a PI3K/AKT-dependent manner. Further supporting this contention, knockdown of PTEN, the natural inhibitor of PI3K/AKT pathway, resulted in up-regulation of PD-L1 that was abolished by the inhibition of AKT [32]. HMGB1 was secreted by melanocytes and keratinocytes upon ultraviolet radiation which subsequently activated RAGE to promote NF-κB and IRF3-dependent transcription of PD-L1 [33]. This is consistent with the current results, which demonstrated that knockdown of RAGE reduced the rHMGB1-mediated upregulation of PD-L1 expression. Similarly, targeting PI3K and AKT in RAGE KD cells could abrogate PD-L1 induction under rHMGB1 treatment.

## Conclusions

Taken together, the data available in the literature along with the data reported herein support the pre-clinical studies to confirm HMGB1-RAGE interaction promoted breast cancer cell invasion via the PD-L1-mediated PI3K/AKT pathway. This literature review confirmed the present study where HMGB1 could regulate breast cancer aggressiveness through RAGE-PI3K/AKT signaling pathway-controlled PD-L1 expression. Mechanically, the attenuation of PD-L1 expression was observed under PI3K and AKT inhibitor treatment. The potential of the attenuation of the HMGB1-RAGE-dependent PI3K/AKT pathway in mediating PD-L1 expression is proposed to both inhibit HMGB1-induced breast cancer aggressiveness and T cell function attenuation (Fig. 5c). The inhibitions of HMGB1 and its related signaling pathways report previously [18] may be proposed to facilitate the efficacy of T cell therapy in breast cancer patients.

## Supplementary Information

**Additional file 1: Figure S1**. Expressions of RAGE, p-AKT and p-ERK.

**Additional file 2: Figure S2**. Cell signaling molecule activation by rHMGB1.

**Additional file 3: Figure S3**. Cell signaling pathway activation by rHMGB1 in MCF-7 cells and MDA-MB-231 BM cells.

**Additional file 4: Figure S4**. Role of HMGB1 on cell proliferation in MDA-MB-231 P by 3-D spheroid-based assay.

**Additional file 5: Figure S5**. Cytotoxicity by sulforhodamine B (SRB) assay performed in MDA-MB-231 P, MDA-MB-231 BM and MCF-7.

**Additional file 6.**

## Data Availability

The datasets used and/or analysed during the current study are available from the corresponding author on reasonable request.

## Abbreviations

AKT: RAC-alpha serine/threonine-protein kinase
ERK: Extracellular signal-regulated kinase
GSK-3: Glycogen synthase kinase 3
HMGB1: High mobility group box 1
JNK: c-Jun N-terminal kinase
KD: Knockdown
mTOR: Mammalian target of rapamycin
MDA-MB-231 BM: MDA-MB-231 bone metastasis variant cell
MDA-MB-231 P: MDA-MB-231 parental cell
RAGE: Receptor for advanced glycation end products
rHMGB1: Recombinant high mobility group box 1
p38: Mitogen-activated protein kinase (MAPK)
PI3K: Phosphoinositide 3-kinase
PD-L1: Programmed death-ligand 1
S6: 40S ribosomal protein S6
STAT: Signal transducer and activator of transcription
