# Supplementary figures and images for: HMGB1 mediates invasion and PD-L1 expression through RAGE-PI3K/AKT signaling pathway in MDA-MB-231 breast cancer cells
Source: BMC Cancer. 2022 May 24;22:578. doi: 10.1186/s12885-022-09675-1 (PMC9128129; doi:10.1186/s12885-022-09675-1)

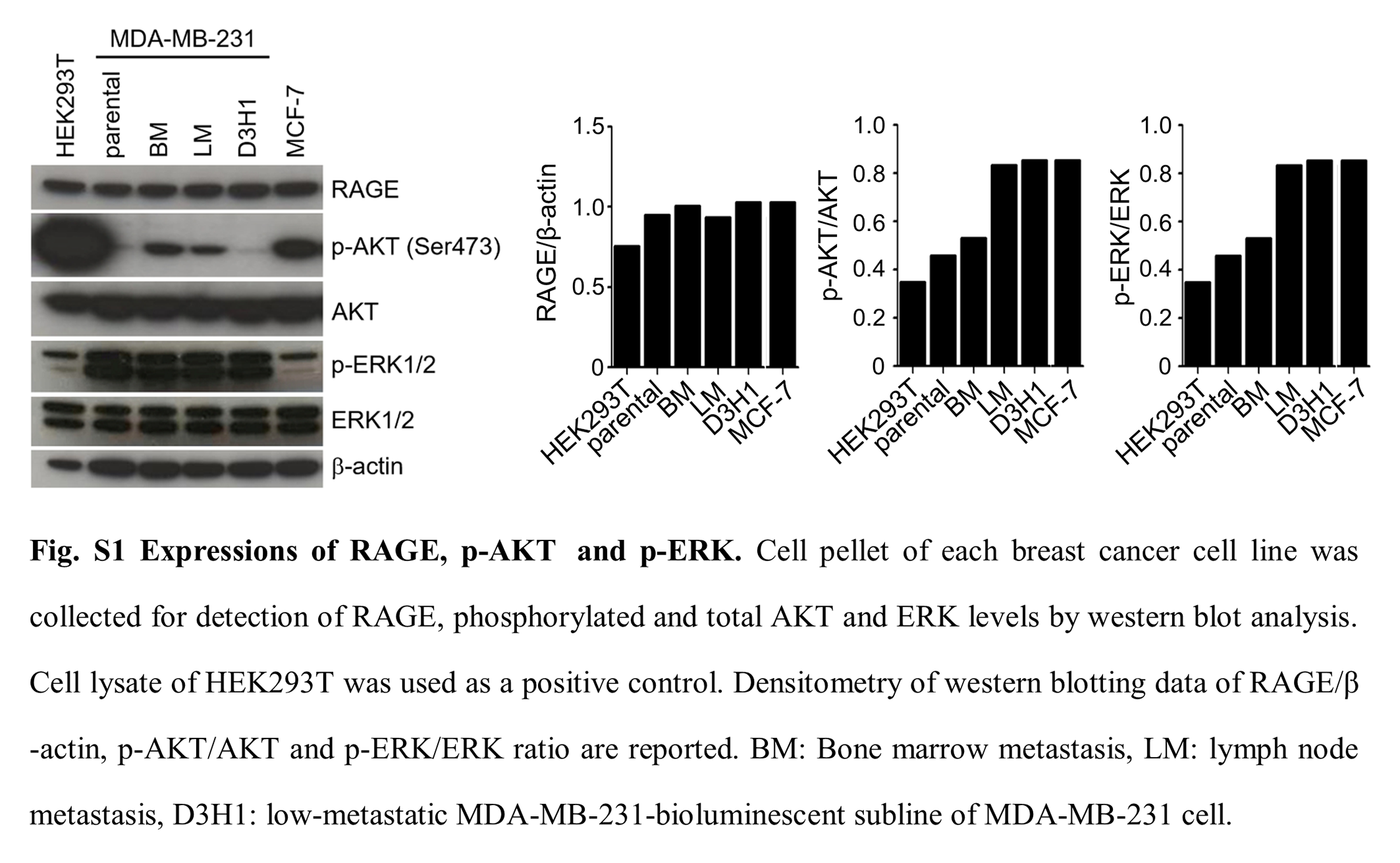

Supplement: Supplementary file 1 — Additional file 1: Figure S1. Expressions of RAGE, p-AKT and p-ERK. [file 12885_2022_9675_MOESM1_ESM.tif]

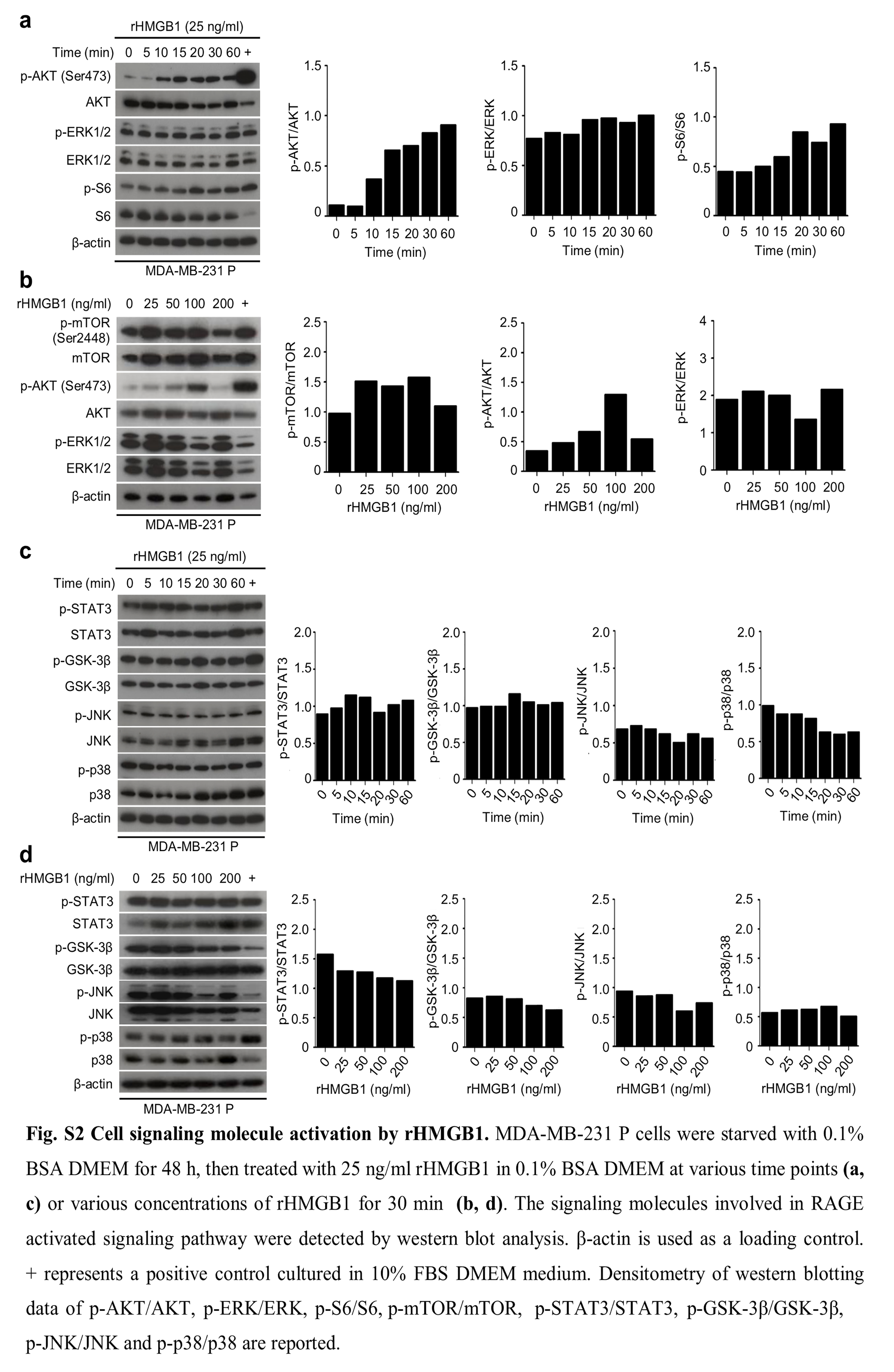

Supplement: Supplementary file 2 — Additional file 2: Figure S2. Cell signaling molecule activation by rHMGB1. [file 12885_2022_9675_MOESM2_ESM.tif]

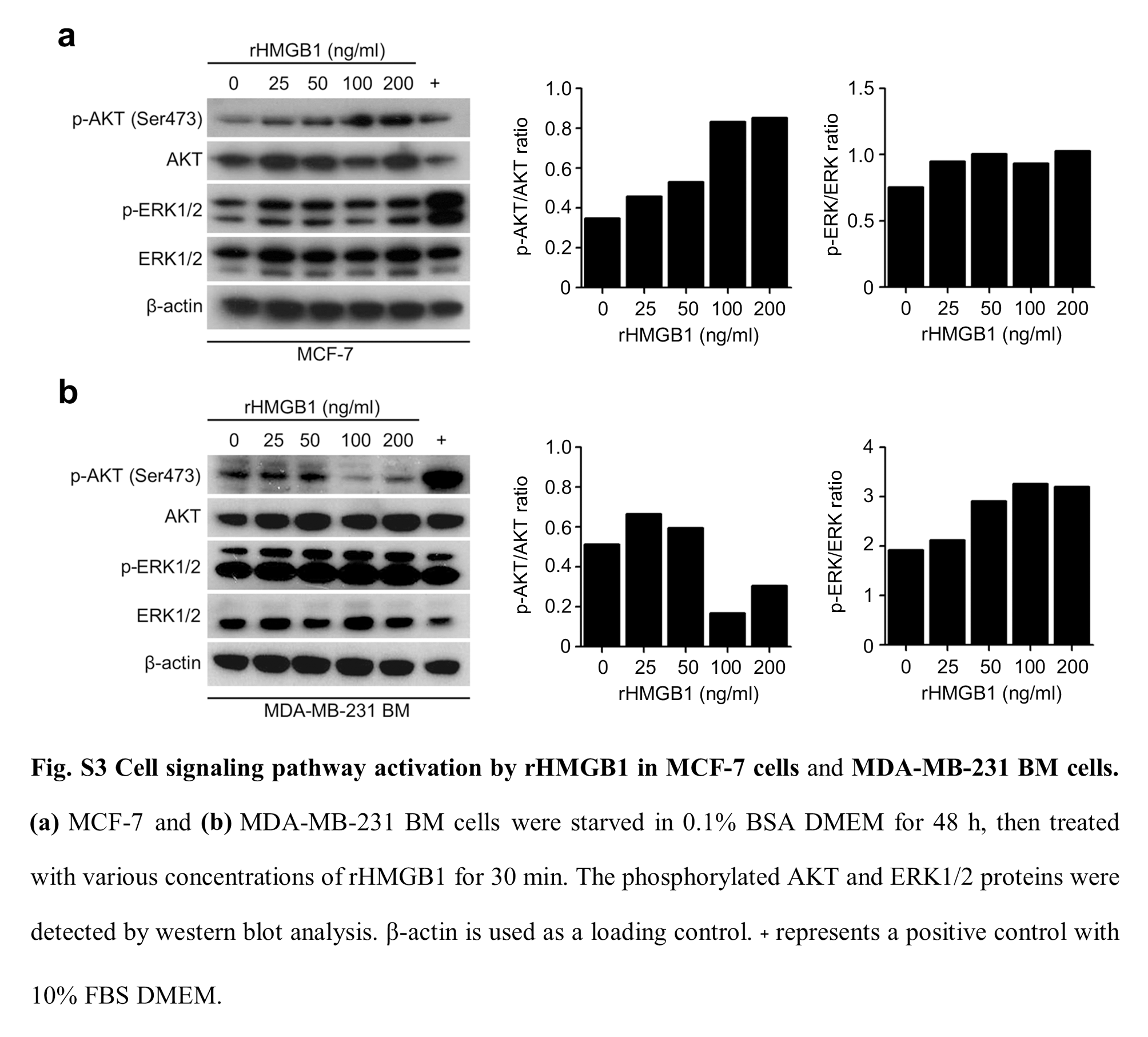

Supplement: Supplementary file 3 — Additional file 3: Figure S3. Cell signaling pathway activation by rHMGB1 in MCF-7 cells and MDA-MB-231 BM cells. [file 12885_2022_9675_MOESM3_ESM.tif]

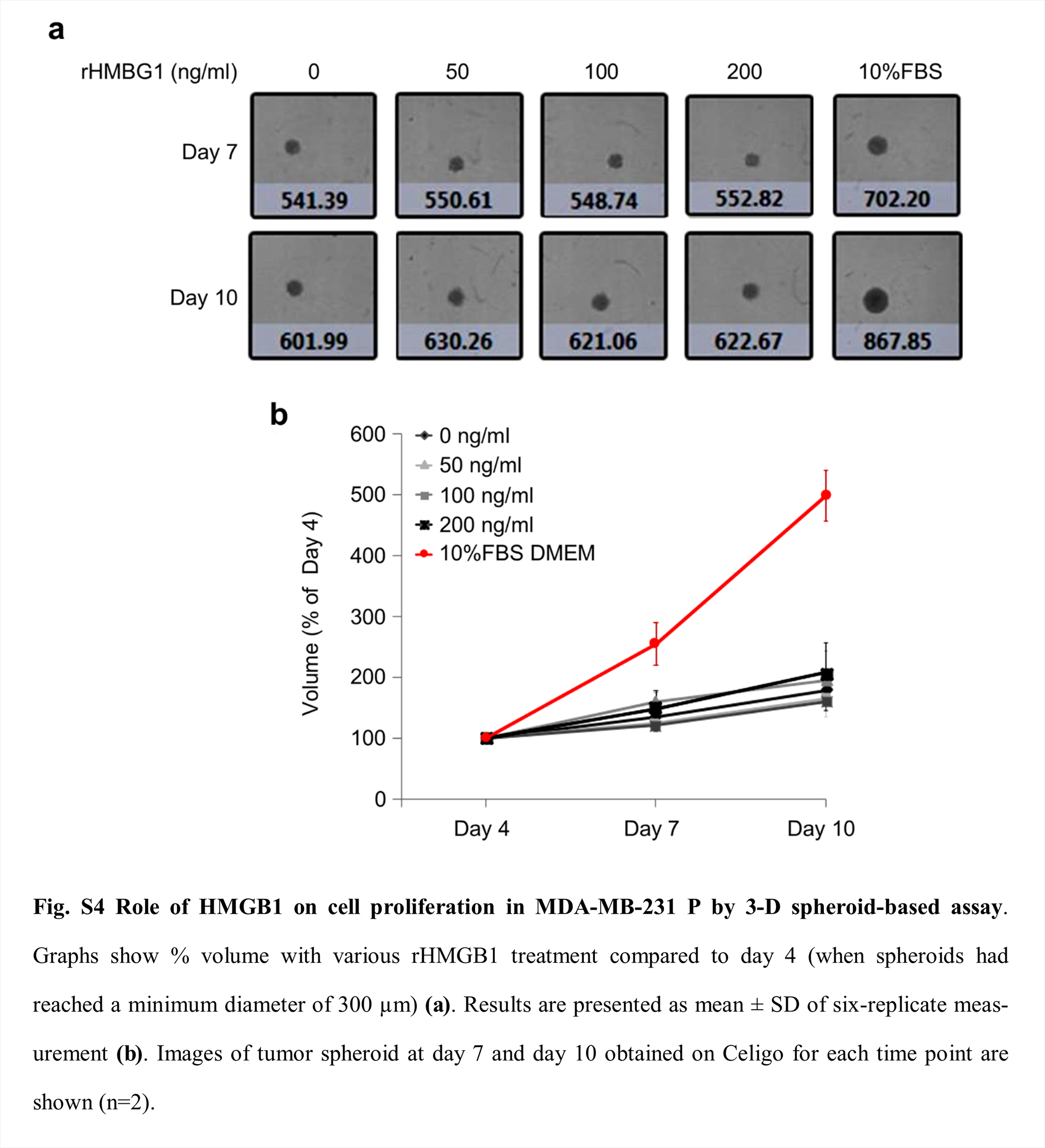

Supplement: Supplementary file 4 — Additional file 4: Figure S4. Role of HMGB1 on cell proliferation in MDA-MB-231 P by 3-D spheroid-based assay. [file 12885_2022_9675_MOESM4_ESM.tif]

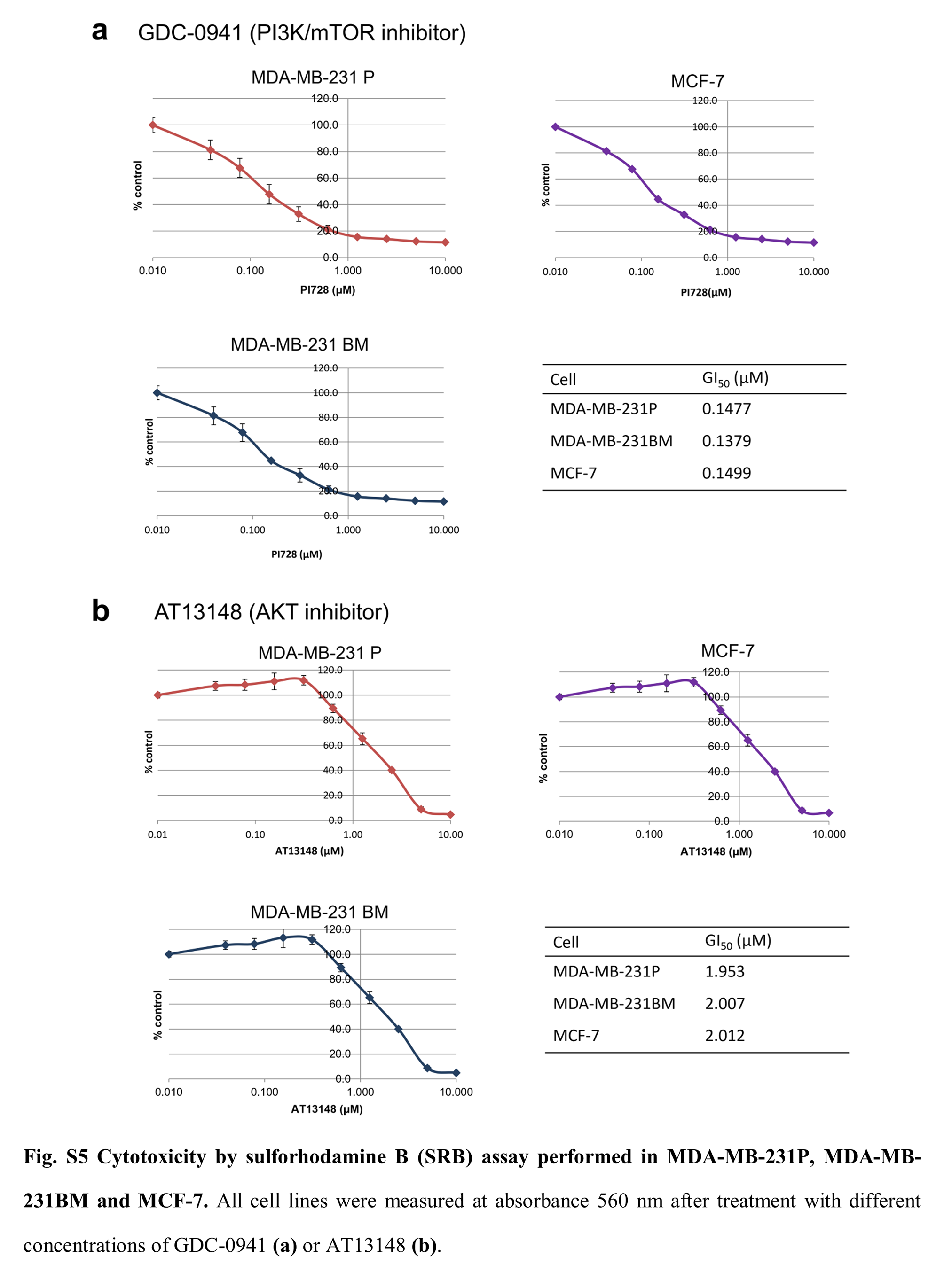

Supplement: Supplementary file 5 — Additional file 5: Figure S5. Cytotoxicity by sulforhodamine B (SRB) assay performed in MDA-MB-231 P, MDA-MB-231 BM and MCF-7. [file 12885_2022_9675_MOESM5_ESM.tif]
