# Supplementary material for: HMGB1 mediates invasion and PD-L1 expression through RAGE-PI3K/AKT signaling pathway in MDA-MB-231 breast cancer cells
Source: BMC Cancer. 2022 May 24;22:578. doi: 10.1186/s12885-022-09675-1 (PMC9128129; doi:10.1186/s12885-022-09675-1)

Uncropped membrane Fig. 3c

RAGE

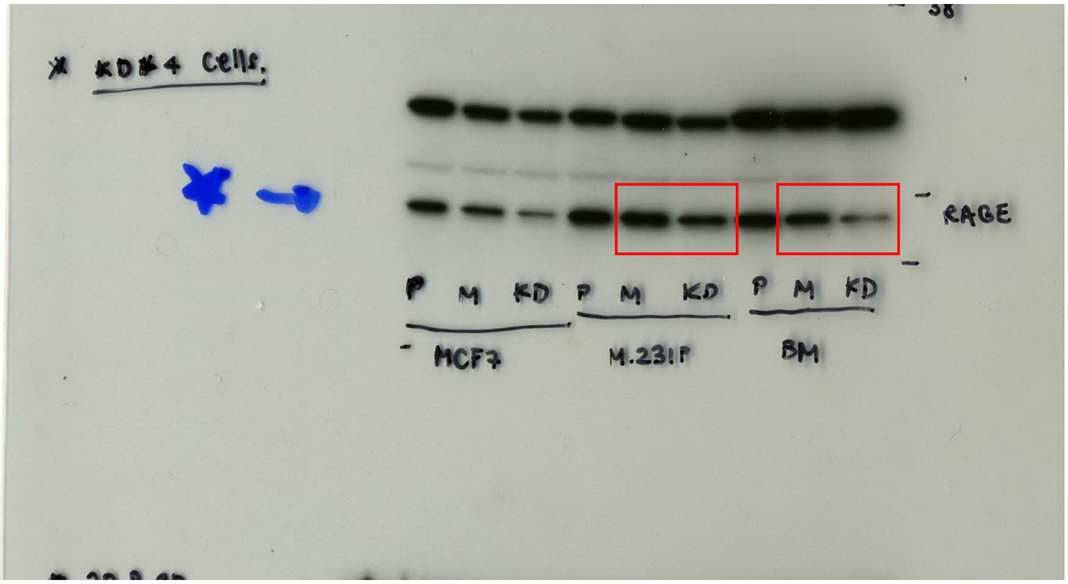

β-actin

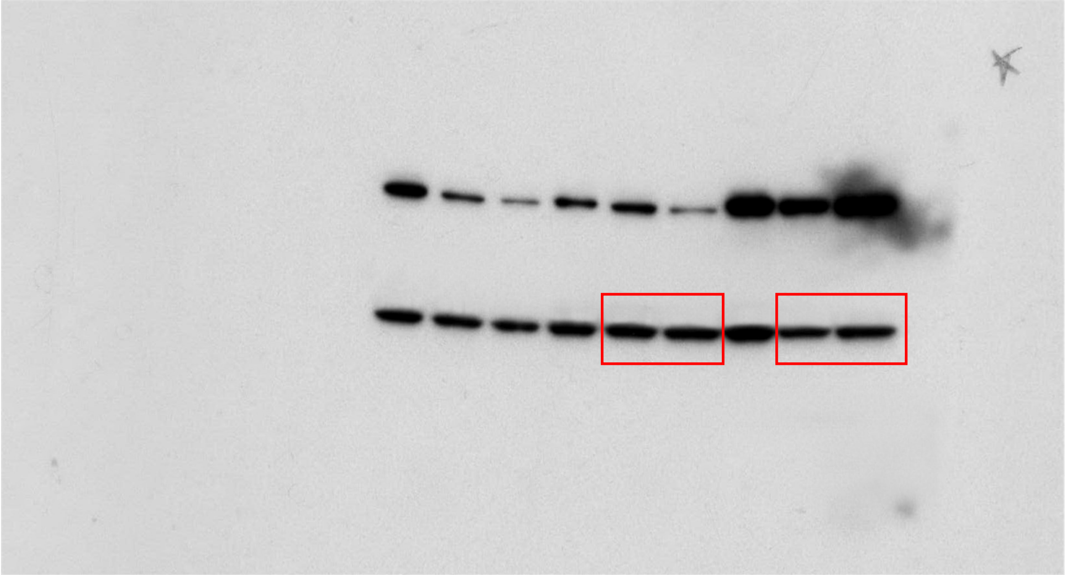

Uncropped membrane Fig. 5a and 5b

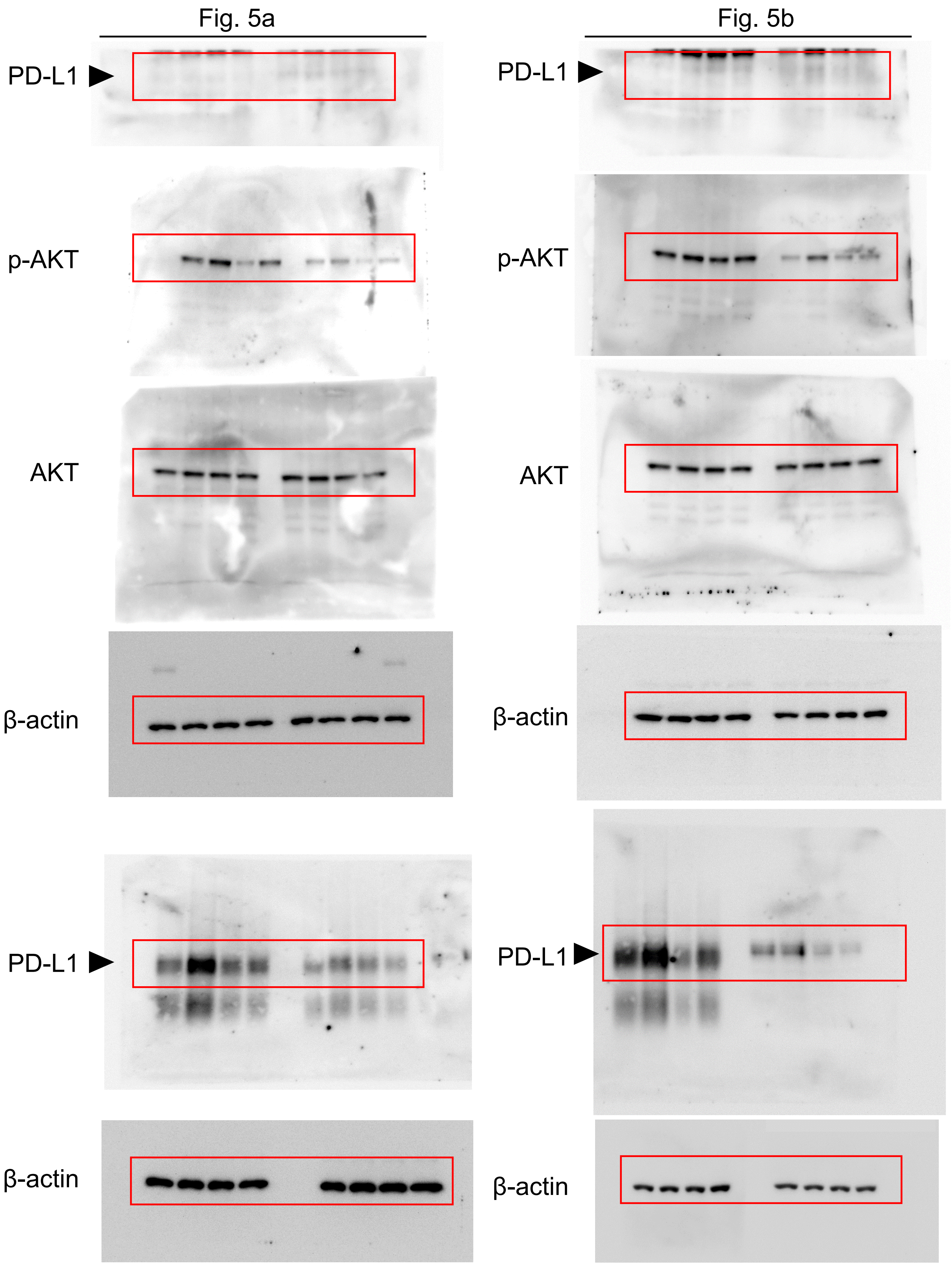

Uncropped membrane Fig. S1

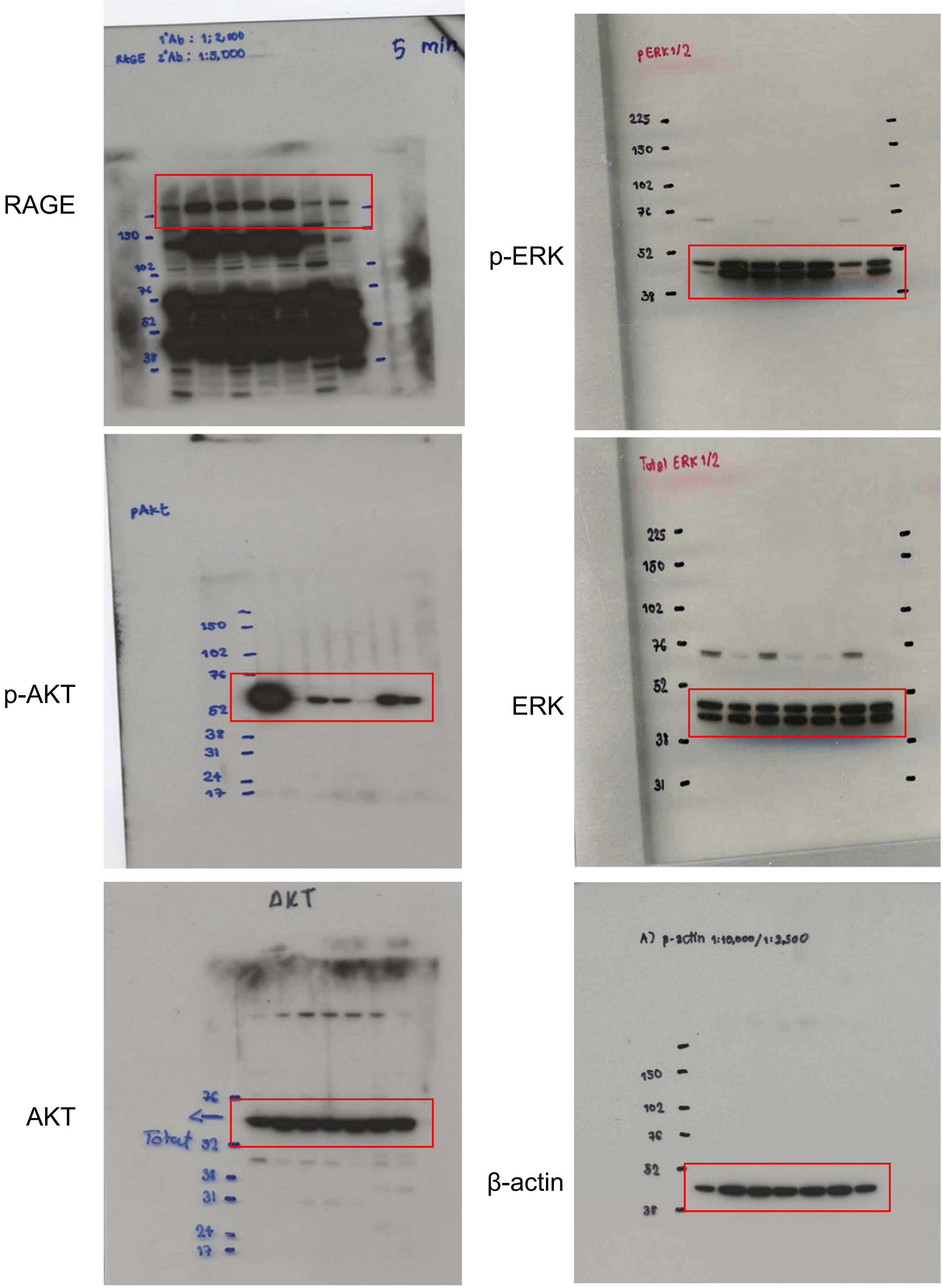

Uncropped membrane Fig. S2a

p-AKT

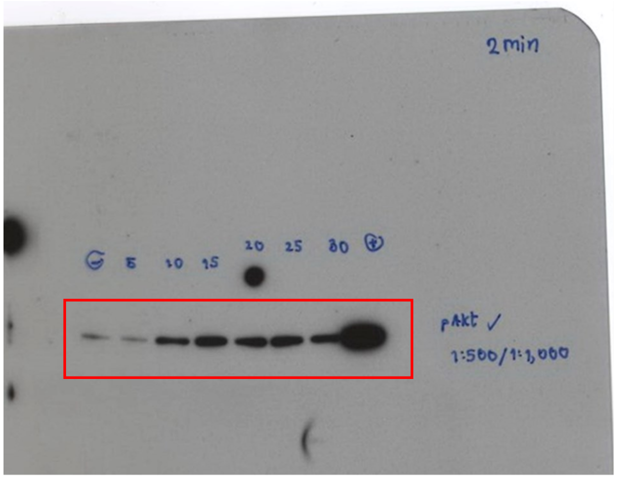

Total AKT

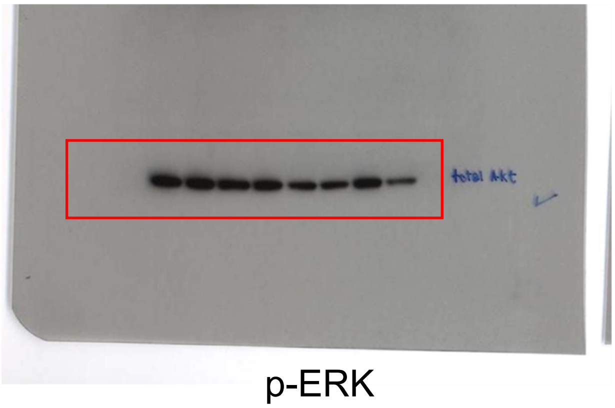

p-ERK

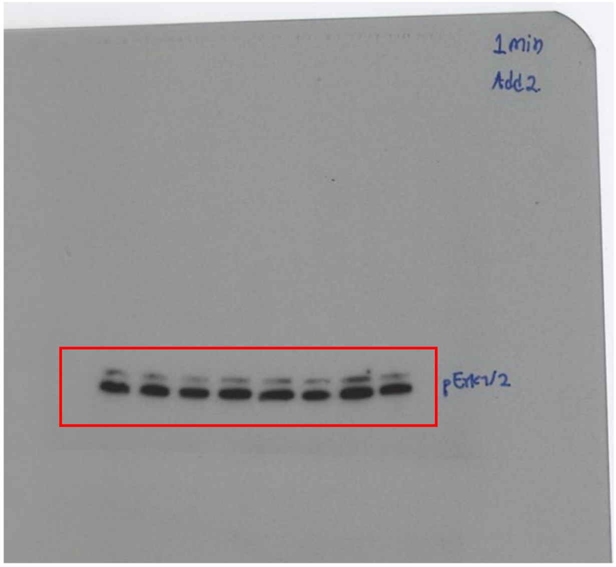

ERK

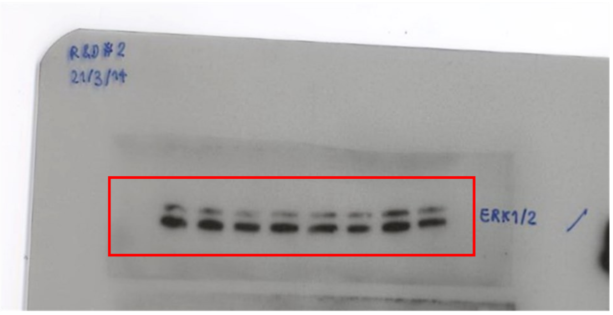

p-S6

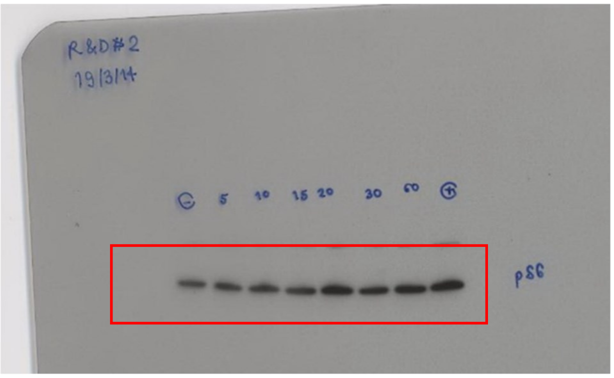

S6

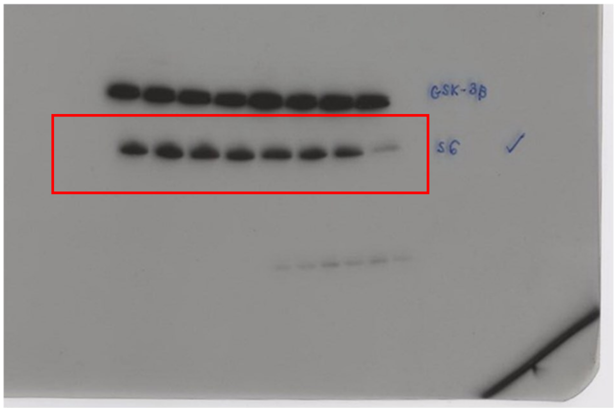

β-actin

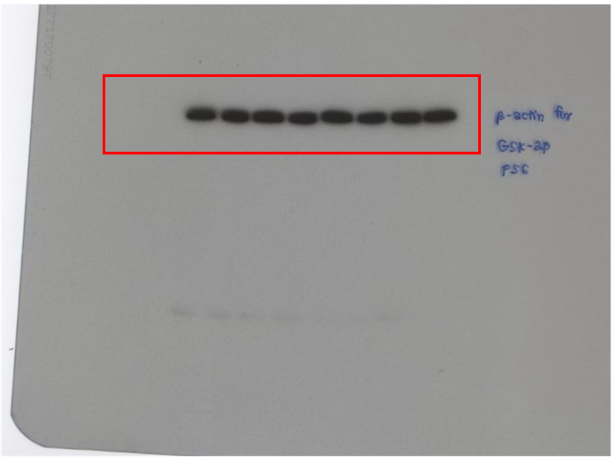

Uncropped membrane Fig. S2b

p-mTOR

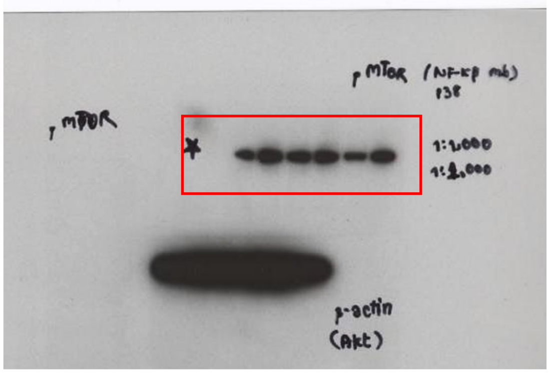

p-ERK

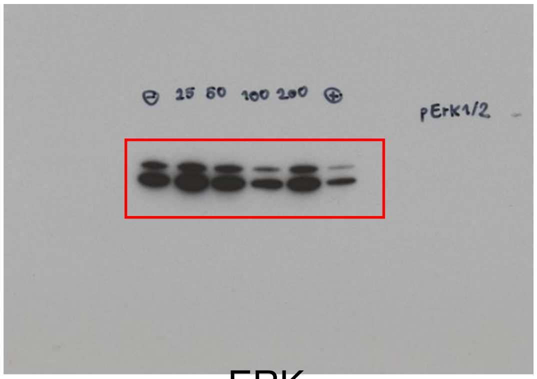

mTOR

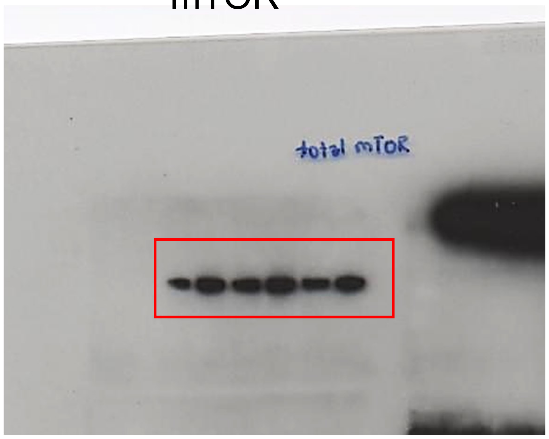

ERK

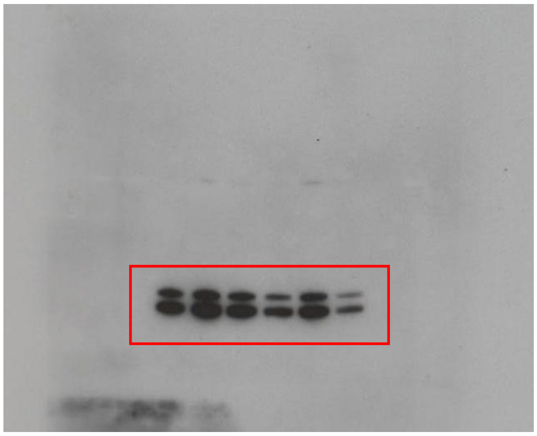

p-AKT

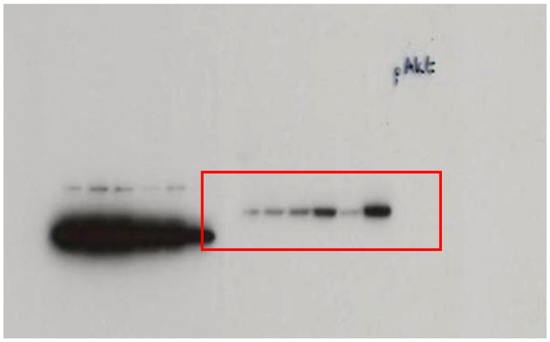

β-actin

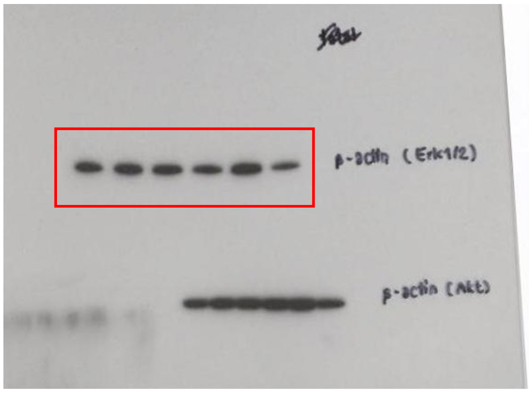

Total AKT

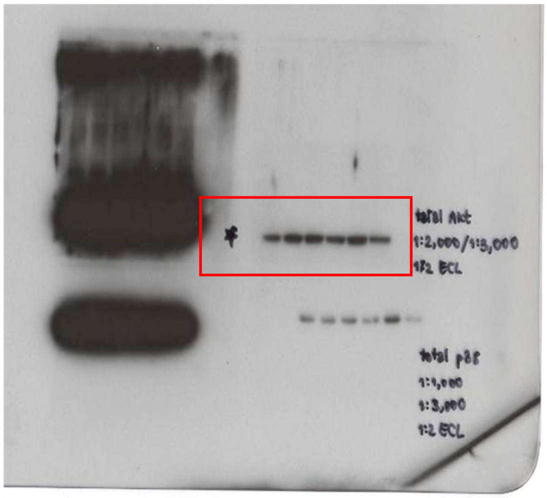

Uncropped membrane Fig. S2c

p-STAT3

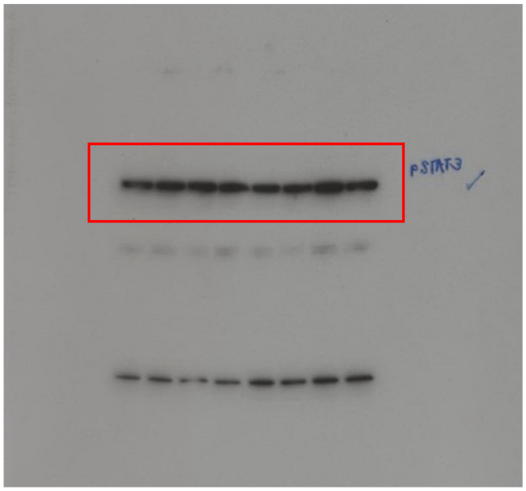

STAT3

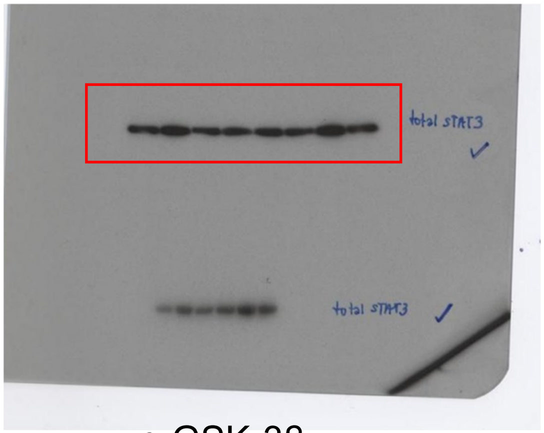

p-GSK-3β

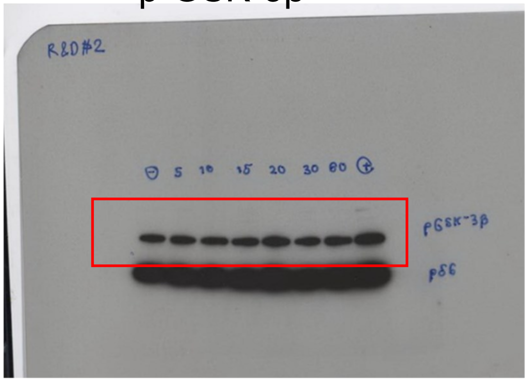

GSK-3β

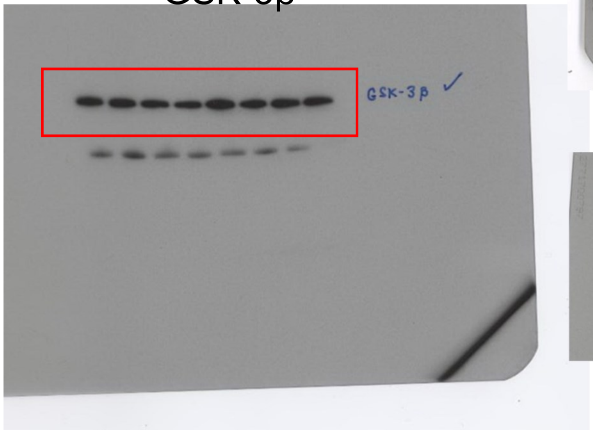

p-JNK

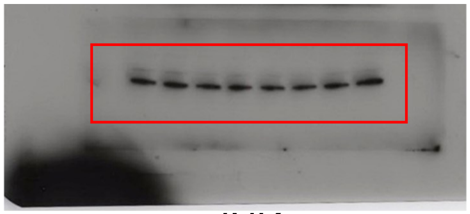

JNK

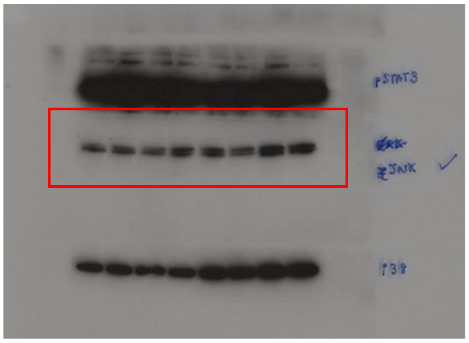

p-p38

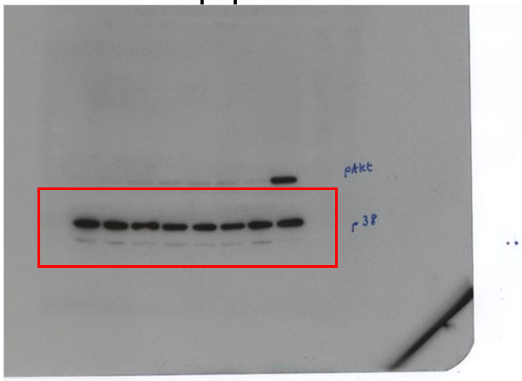

p38

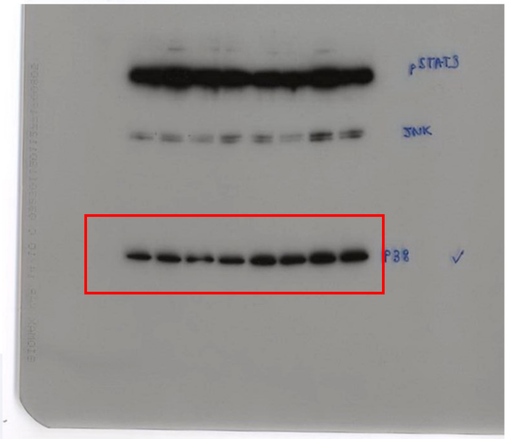

β-actin

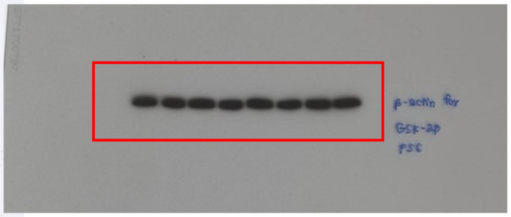

Uncropped membrane Fig. S2d

p-STAT3

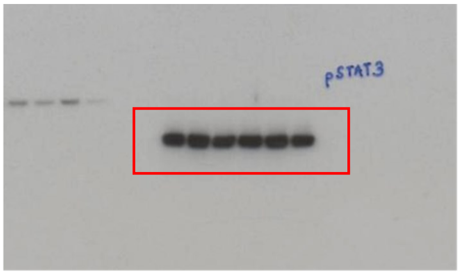

STAT3

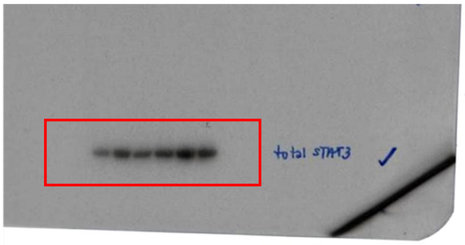

p-GSK-3β

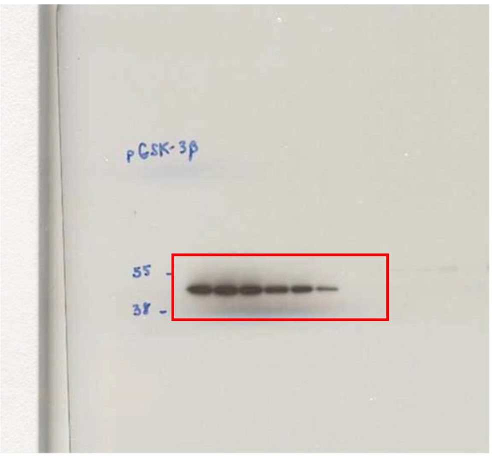

GSK-3β

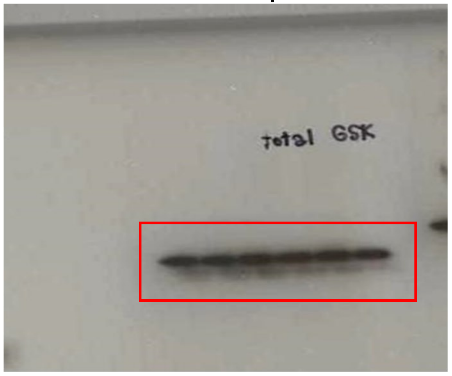

β-actin

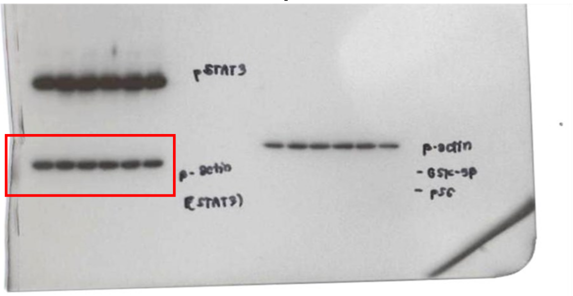

p-JNK

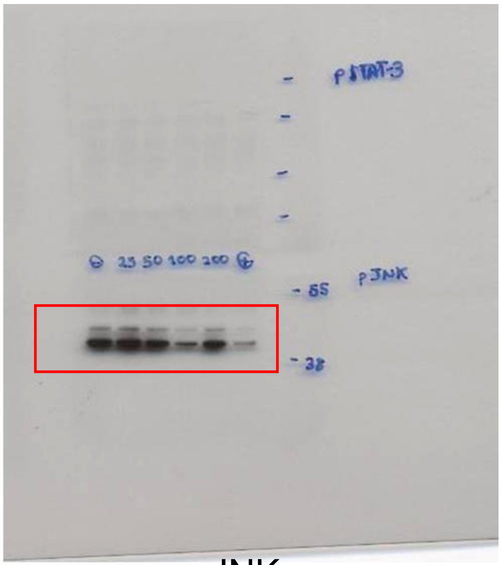

JNK

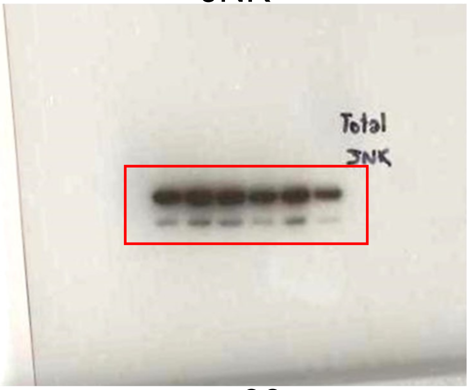

p-p38

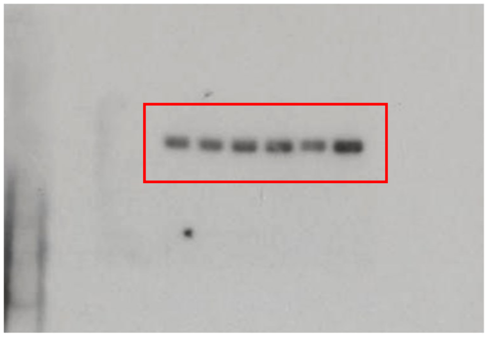

p38

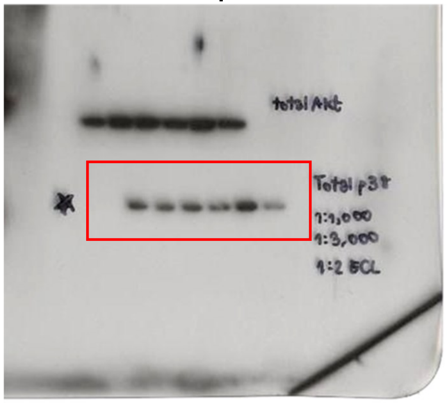

Uncropped membrane Fig. S3a

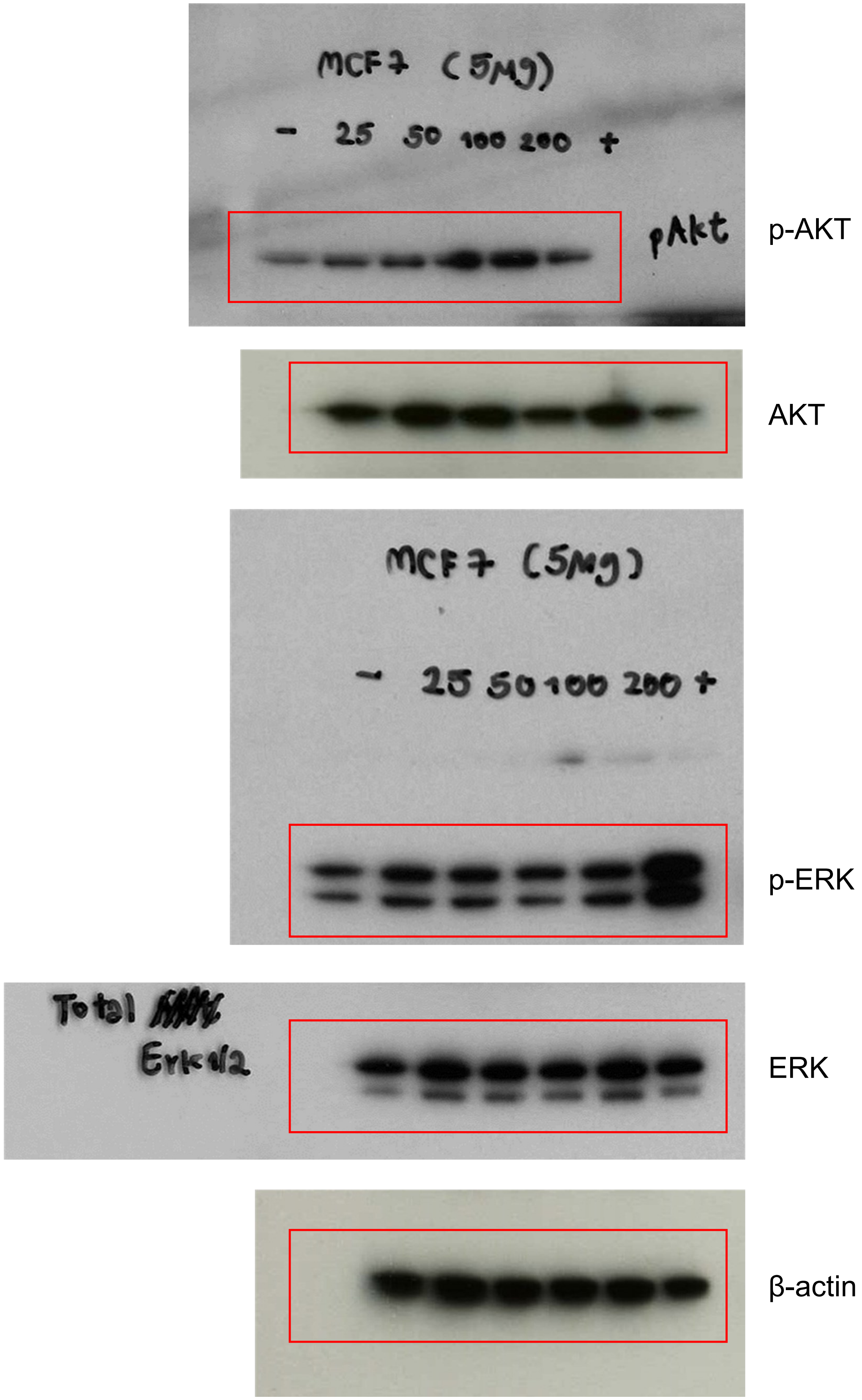

Uncropped membrane Fig. S3b

p-AKT

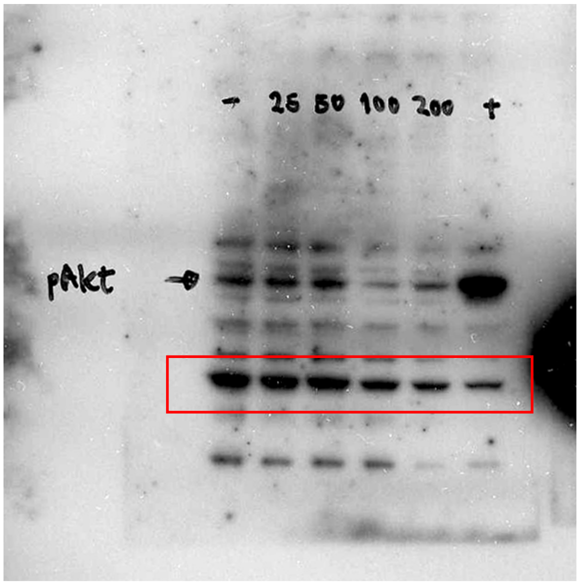

p-ERK

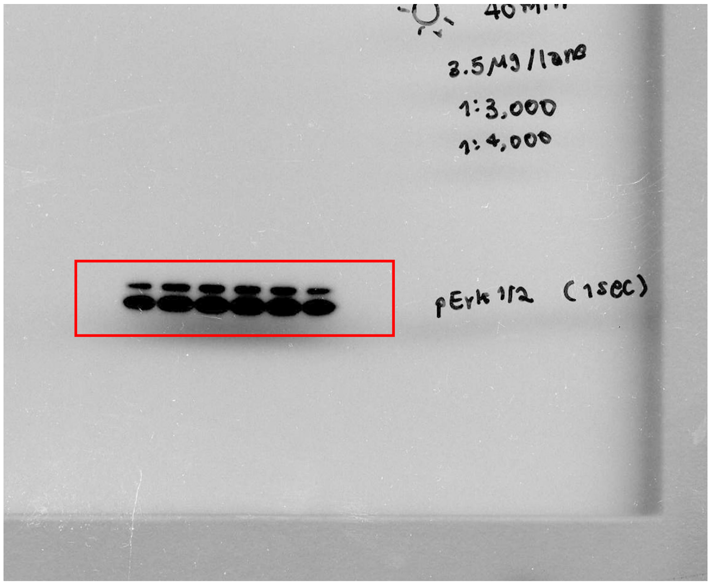

AKT and β-actin

AKT

β-actin

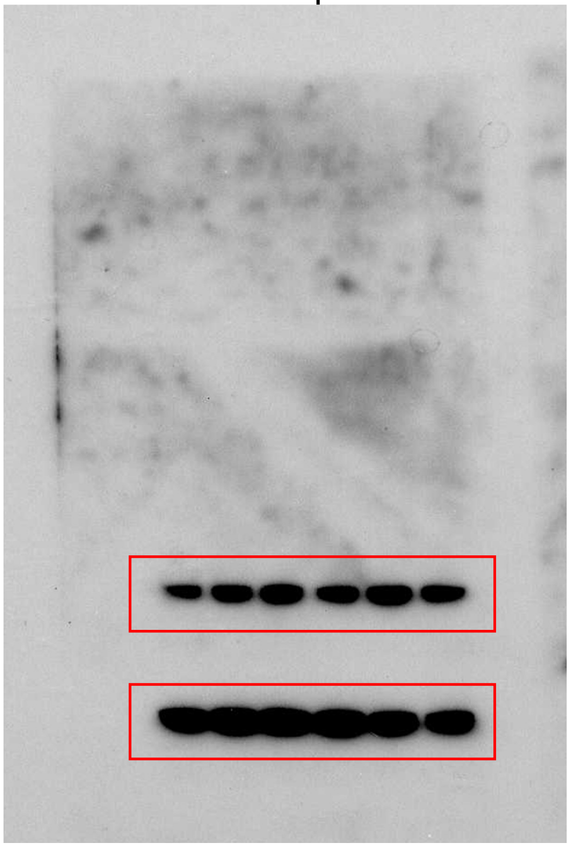

ERK

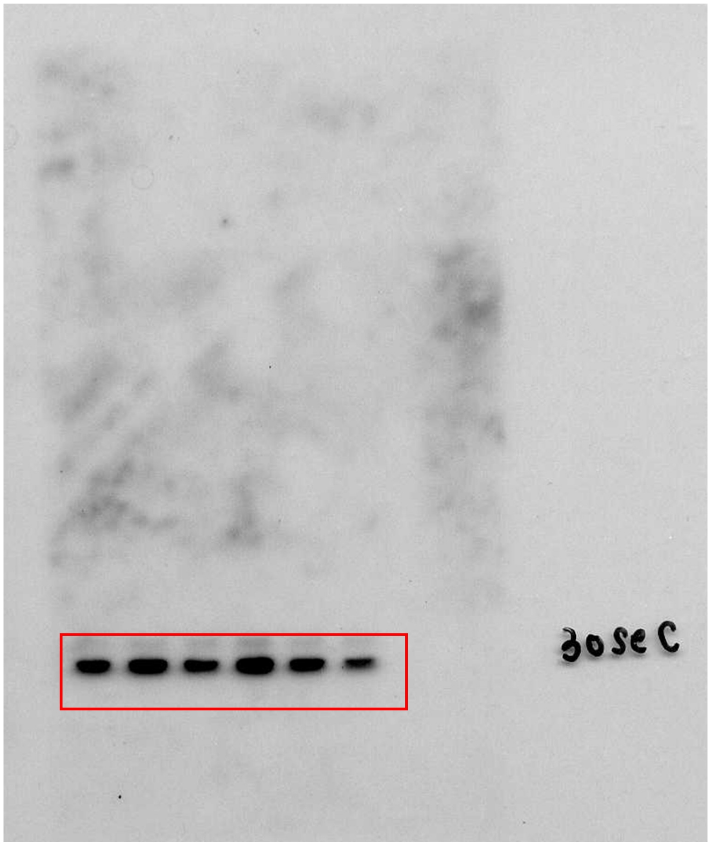

Supplement: Supplementary file 6 — Additional file 6. [file 12885_2022_9675_MOESM6_ESM.pdf]
